# Therapeutic and prophylactic activity of itraconazole against human rhinovirus infection in a murine model

**DOI:** 10.1038/srep23110

**Published:** 2016-03-15

**Authors:** Aeri Shim, Jae-Hyoung Song, Bo-Eun Kwon, Jeong-Jun Lee, Jae-Hee Ahn, Yeon-Jeong Kim, Ki-Jong Rhee, Sun-Young Chang, Younggil Cha, Yong-Soo Lee, Mi-Na Kweon, Kwi Sung Park, Dong-Eun Kim, Sungchan Cho, Hyun-Jong Cho, Hyun-Jeong Ko

**Affiliations:** 1 College of Pharmacy, Kangwon National University, Chuncheon, South Korea; 2 College of Pharmacy, Inje University, Gimhae, South Korea; 3 Department of Biomedical Laboratory Science, Yonsei University, Wonju, South Korea; 4 College of Pharmacy, Ajou University, Suwon, South Korea; 5 University of Ulsan College of Medicine, Asan Medical Center, Seoul, South Korea; 6 Chungcheongnam-do Institute of Health and Environmental Research, Daejeon, South Korea; 7 Incurable Diseases Therapeutics Research Center, Korea Research Institute of Bioscience & Biotechnology, Cheongju, South Korea

## Abstract

Human rhinovirus (HRV) is the most common viral infectious agent in humans and is the predominant cause of the common cold. There is a need for appropriate vaccines or therapeutic agents to treat HRV infection. In this study, we investigated whether itraconazole (ICZ) can protect cells from HRV-induced cytotoxicity. Replication of HRV1B was reduced by ICZ treatment in the lungs of HRV1B- as compared to vehicle-treated mice. The numbers of immune cells, including granulocytes and monocytes, were reduced in bronchoalveolar lavage fluid (BALF) by ICZ administration after HRV1B infection, corresponding to decreased pro-inflammatory cytokine and chemokine levels in BALF. A histological analysis of lung tissue showed that ICZ suppressed inflammation caused by HRV1B infection. Interestingly, pretreatment of mice with ICZ in the form of a nasal spray had potent prophylactic antiviral activity. Cholesterol accumulation in the plasma membrane was observed upon HRV infection; ICZ blocked cholesterol trafficking to the plasma membrane, as well as resulted in its accumulation in subcellular compartments near the nucleus. These findings suggest that ICZ is a potential antiviral agent for the treatment of HRV infection, which can be adopted preventatively as well as therapeutically.

Human rhinoviruses (HRVs) are members of the *Enterovirus* genus of the family *Picornaviridae* and have a positive-sense, single-stranded RNA genome of approximately 7200 bp^1^. Infection by HRVs is a major cause of the common cold, which can be accompanied by sore throat and ear or sinus infection and can lead to pneumonia and bronchiolitis^2^. HRVs usually infect the upper respiratory tract^3^,^4^, but can also lead to the exacerbation of asthma^5^. In addition, individuals with allergies are more susceptible to HRV infection, which is usually complicated by severe lower respiratory tract inflammation^6^. Interestingly, defects in innate immunity (e.g., type III interferon-λ production) have been reported in response to HRV infection in asthmatic patients^7^, underscoring the necessity for the development of antiviral drugs to treat HRV infection. However, owing to the genomic instability of HRVs and limitations of animal models, there have been no vaccines or antiviral agents approved for the prevention or treatment of HRV infection. In addition, clinical trials for antiviral therapies have been limited by toxicity, drug interactions, and the possible emergence of drug-resistant mutant virus strains^8^.

Itraconazole (ICZ) belongs to the triazole group of antifungals with potent lipophilic features, low toxicity, and broad-spectrum antifungal activity against pathogenic yeasts and fungi^9^,^10^. ICZ is orally administered as a drug for *Cryptococcus neoformans*, *Histoplasma capsulatum*, and several other opportunistic fungal pathogens and is sometimes given as a nasal spray. ICZ has anti-viral effects against human immunodeficiency virus^11^,^12^,^13^ and hepatitis^14^,^15^,^16^, but there have been no animal studies investigating the antiviral activity of ICZ against HRVs.

ICZ acts by inhibiting fungus-induced ergosterol biosynthesis, but is otherwise pharmacologically distinct from other triazole agents. Recent studies suggest that ICZ inhibits the proliferation of several types of cancer cell in an autophagy-dependent manner via disruption of cholesterol trafficking, resulting in reduced plasma membrane cholesterol content^17^. ICZ also exhibits antiangiogenic activity that is exerted via blockade of cholesterol trafficking through the lysosome, leading to mammalian target of rapamycin (mTOR) inhibition in endothelial cells; mTOR inhibition by ICZ was partially restored by adding extracellular cholesterol or by thapsigargin treatment^18^.

Recent studies have shown that cholesterol-enriched membrane domains are required in cells infected by HRV despite the presence of receptors for HRV entry such as intercellular adhesion molecule (ICAM)-1 and low-density lipoprotein (LDL) receptor^19^,^20^. Interestingly, pretreatment of human cells with cyclodextrin (CD) decreased HRV infection rate and was associated with a reduction in cholesterol-enriched membrane rafts^19^,^21^.

In the current study, we identified ICZ as a candidate antiviral agent against HRV infection in a screen of the Food and Drug Administration (FDA)-approved drug library. We confirmed the antiviral activity of ICZ in a mouse model of HRV1B infection, and found that ICZ acts as a cholesterol trafficking inhibitor that reduces membrane cholesterol levels.

## Results

### ICZ has antiviral activity against HRV1B *in vitro*

**Figure 1:**
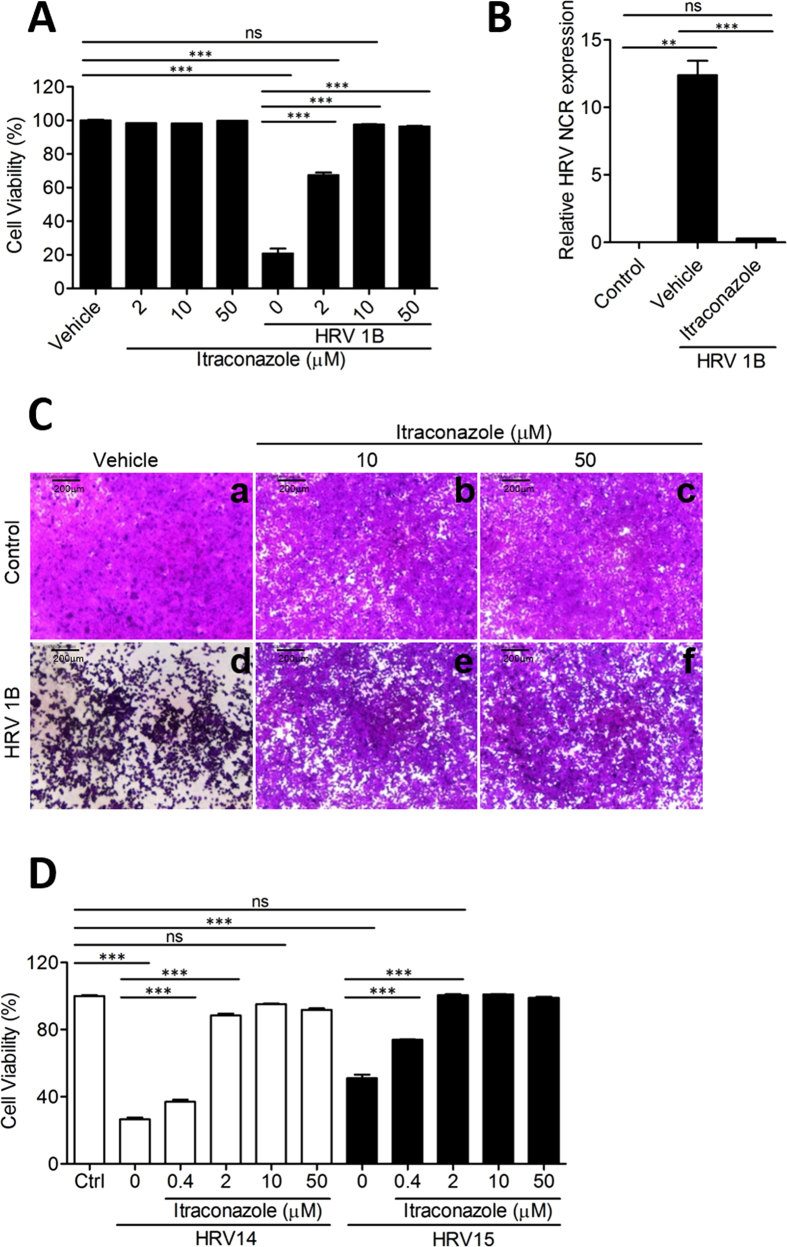
Itraconazole has antiviral activity against HRV *in vitro*. (**A**) Cell viability was evaluated based on results of the SRB assay. HeLa cells were infected at the 50% cell culture infective dose (CCID_50_) of HRV1B and treated with indicated concentrations of ICZ. Cell viability was determined based on absorbance at 520 nm. (**B**) Relative HRV gene expression in control, HRV1B-infected, and 10 μm ICZ-treated cells by real-time PCR. (**C**-**a**) Control cells; (**C**-**b**) 10 μm and (**C-c**) 50 μm ICZ-treated cells; (**C-d**) HRV1B-infected cells; (**C**-**e**) 10 μm and (**C**-**f**) 50 μm ICZ-treated cells infected with HRV1B. (**D**) Cell viability was evaluated based on results of the SRB assay. HeLa ells were infected at the CCID_50_ of HRV14 and HRV15 and treated with indicated concentrations of ICZ. Cell viability was determined based on absorbance at 520 nm. Bar graphs show mean ± SEM. ^∗∗^P < 0.01, ^∗∗∗^P < 0.001, Newman-Keuls multiple comparisons test (analysis of variance).

In a screen of the FDA library to identify candidate antiviral drugs against HRV, we found that several compounds including ketoconazole, ICZ, terconazole, and posaconazole enhanced viability in HeLa cells infected with HRV1B when used at a concentration of 10 μM (Fig. S1). The antiviral effect of ICZ against HRV1B was dose-dependent as determined by the sulforhodamine B (SRB) assay, with significant antiviral activity at a concentration of 2 μM that was enhanced at concentrations of 10 and 50 μM. ICZ was not toxic to HeLa cells; viability was about 100% at concentrations between 2 and 50 μM (Fig. 1A). Other azole agents such as posaconazole (Fig. S1B), ketoconazole (Fig. S1C), and terconazole (Fig. S1D) also exhibited low cytopathic effect (CPE), but were less effective in terms of protection against HRV1B infection than ICZ. Furthermore, terconazole and ketoconazole were toxic to HeLa cells at high concentrations (50 μM) (Fig. S1C,D). We therefore examined the antiviral activity of ICZ against HRV in greater detail.

To determine whether the increase in cell viability upon ICZ treatment in HRV1B-infected cells was due to direct antiviral activity of the drug, we assessed HRV1B 5′ non-coding region (NCR) mRNA level 48 h after infection. The expression of 5′ NCR transcript was decreased by ICZ (10 μM) as compared to vehicle treatment (Fig. 1B), suggesting that HRV1B replication was inhibited by ICZ. To further confirm the active infection of HRV1B in cell culture could be inhibited by ICZ treatment, we used strand-specific probes for HRV1B. The cultured cells infected with HRV1B were harvested at various time points (2, 4, 6, 8, 10, 12, 14, and 16 h post-infection), and the content of virus RNAs was analyzed using RT-PCR. We could detect the positive strand RNA of HRV1B virus starting at 8 h postinfection and the negative strand of RNA was obviously seen at 10 h post-infection, albeit the level was relatively low as compared to that of positive strand RNA (Fig. S2). Interestingly, ICZ effectively inhibited replication of HRV1B similar to Rupintrivir and Pleconaril which has been well-known as HRV inhibitors.

We also assessed morphological changes in HeLa cells by microscopy (Fig. 1C). Uninfected control cells attached to the culture dish; severe cytopathic effects were observed 2 days after HRV1B infection (Fig. 1C). HeLa cells treated with 10 and 50 μM ICZ without HRV1B infection showed typical flattened shapes and normal morphology; HRV1B-infected cells treated with 10 and 50 μM ICZ survived and there was no CPE observed (Fig. 1C).

ICZ as well as other azole agents had anti-viral effect against HRV14 and HRV15 (Fig. 1D, Fig. S3,4), which exploit ICAM-1 as a viral receptor^22^,^23^; HRV1B uses LDL as a viral receptor^24^, suggesting that ICZ has non-specific antiviral activity against HRVs. ICZ also showed antiviral activity against CVB3 and EV71 of the *Picornaviridae* family, but not against influenza virus [A/PR/8/1934(H1N1)] of the *Orthomyxoviridae* family (Fig. S5–7), indicating that ICZ is effective against a broad spectrum of *Picornaviridae* family viruses.

### Oral administration of ICZ reduces HRV1B replication in mice

**Figure 2:**
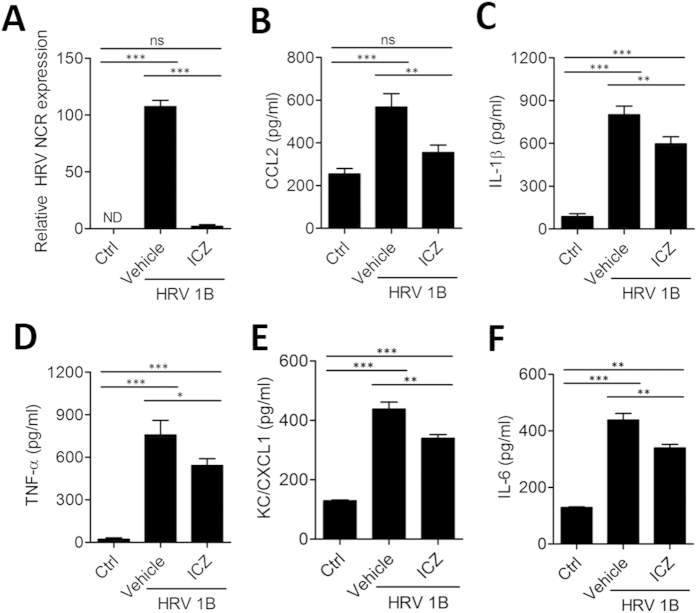
Oral administration of ICZ attenuates pulmonary cytokine/chemokine production in HRV-infected mice. Mice were orally administered 20 mg/kg ICZ. Cytokine/chemokine levels were assessed in the supernatant of lung tissue homogenates from mice 8 h after HRV1B infection. (**A**) Relative HRV gene expression levels in the lung, and (**B–F**) levels of CCL2, IL-1β, TNF-α, KC, and IL-6 were measured. Data represent mean ± SEM (n = 6). ^∗^P < 0.05, ^∗∗^P < 0.01, ^∗∗∗^P < 0.001, Newman-Keuls multiple comparisons test (analysis of variance).

To assess the antiviral activity of ICZ against HRV1B *in vivo*, mice were orally administered 20 mg/kg ICZ 1 h prior to and 4 h after intranasal HRV1B infection. Mice were infected with a placebo and HRV1B-infected mice were orally administered vehicle as negative controls. Lung tissue samples were obtained from the mice and total RNA was extracted from lung tissue in order to assess viral gene expression. We found that HRV1B expression peaked at 8 h and inflammatory cytokines were produced at 24 h post-infection (Fig. S8)^25^. To further confirm that active replication of HRV1B occurs in the lungs of infected mice after intranasal infection, we decided to detect the negative strand of HRV1B RNA by RT-PCR using specific probes. The existence of negative strand RNA as well as positive strand RNA was detected at 8 h post-infection, suggesting active HRV replication in mice. In the lung tissues of mice intranasally inoculated with UV-treated HRV1B virus, we could not detect positive or negative strand of HRV1b RNA (Fig. S9). At 8 h post-infection, ICZ treatment effectively inhibited the expression of positive- and negative-strand HRV1B RNA in mice as compared to vehicle treatment (Fig. 2A and S9). These results confirm that ICZ has anti-HRV activity *in vivo* when administered systemically via the oral route.

### ICZ treatment reduces pulmonary cytokine/chemokine production in HRV-infected mice

**Figure 3:**
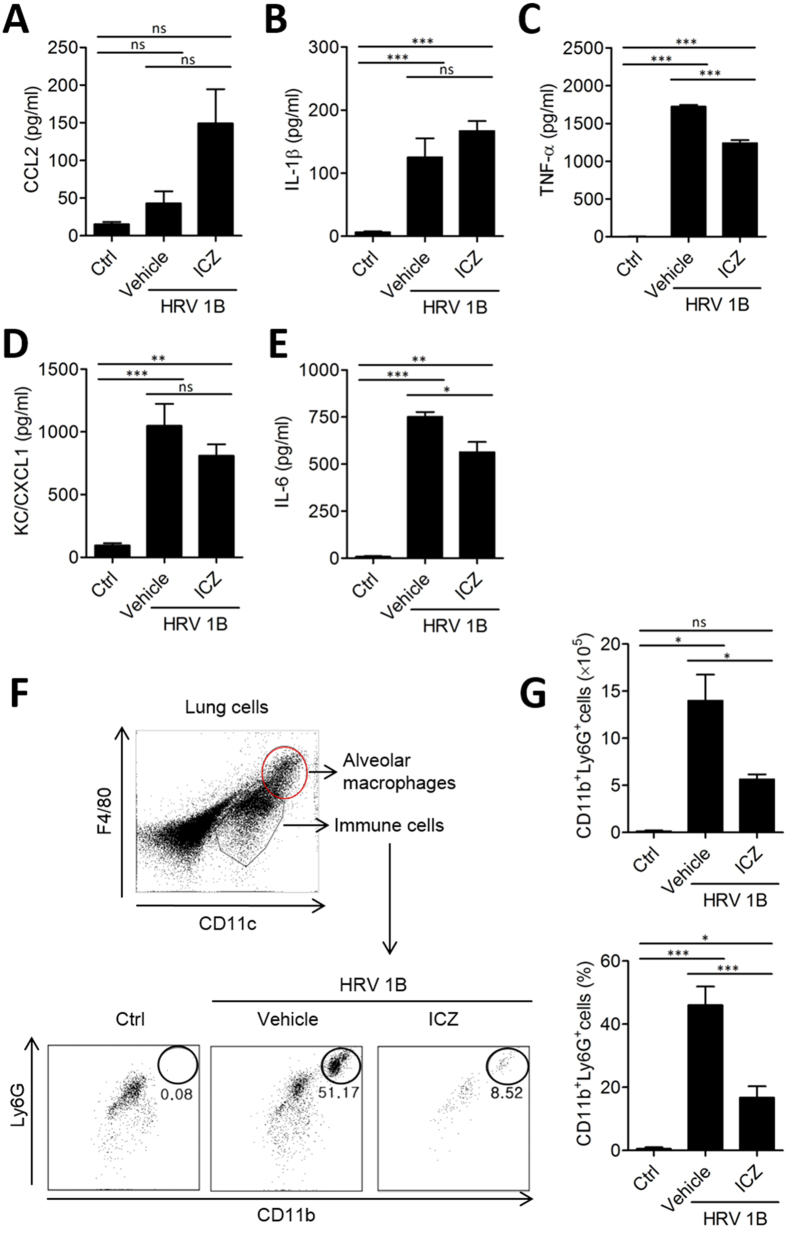
ICZ treatment reduces infiltration of inflammatory cells in the BALF of HRV-infected mice. (**A–E**) Cytokine and chemokine levels in BALF of HRV1B-infected mice. (**F**) Percentage of neutrophils in BALF, as determined by flow cytometry after gating out CD11c^high^F4/80^high^ alveolar macrophages. (**G**) Number and percentage of CD11b ^+^ Ly6G ^+^ cells in BALF. Data represent mean ± SEM (n = 4). ^∗^P < 0.05, ^∗∗^P < 0.01, ^∗∗∗^P < 0.001, Newman-Keuls multiple comparisons test (analysis of variance).

Virus-induced cytokines and chemokines, including chemokine (C-X-C motif) ligand (CXCL)1/keratinocyte chemoattractant (KC), chemokine (C-C motif) ligand (CCL)2, interleukin (IL)-6, IL-1β, and tumor necrosis factor (TNF)-α are secreted into alveolar spaces and bronchoalveolar lavage fluid (BALF) 8 h after HRV1B infection and activate innate immune responses that induce an acute inflammatory response^25^,^26^,^27^. To determine the effect of ICZ on pulmonary cytokine and chemokine secretion after HRV infection, mice were treated with 20 mg/kg ICZ 1 h before and 4 h after intranasal HRV1B infection. At 8 h post-infection, cytokine and chemokine levels in the lungs and BALF were measured by enzyme-linked immunosorbent assay (ELISA). CCL2, IL-1β, TNF-α, CXCL1/KC, and IL-6 levels in the lungs were increased upon HRV1B infection, and there was significant reduction in the levels of those cytokines and chemokines by ICZ treatment (Fig. 2B–F). In the BALF, TNF-α and IL-6 levels were elevated upon HRV1B infection and were reduced by ICZ treatment; CCL2, IL-1β, and CXCL1/KC levels were not significantly altered (Fig. 3), while cytokine mRNA levels in the lungs were decreased (Fig. S10). These results suggest that increases in pro-inflammatory cytokine and chemokine levels induced by HRV infection were mitigated by ICZ treatment, which may be associated with reduced viral load.

### Cell infiltration into the bronchoalveolar space after HRV infection is diminished by ICZ treatment

To further assess the effects of ICZ treatment on bronchoalveolar inflammation induced by HRV infection, we evaluated cell infiltration in BALF of HRV1B-infected mice with or without ICZ treatment. Specifically, we examined whether neutrophils—the primary innate immune cells that respond to HRV infection—were present (Fig. 3F,G). CD11c^high^F4/80^high^ alveolar macrophages were excluded from the gating of neutrophils. The number of CD11b^+^Ly6G^+^ cells in the BALF exhibiting the granulocytic neutrophil phenotype was increased in HRV-infected as compared to uninfected mice. The relative numbers of these cells was decreased by ICZ treatment (Fig. 3G), confirming that inflammatory cells—especially neutrophils—in lungs were increased by HRV1B infection, which was significantly inhibited by administration of ICZ.

### HRV-induced lung inflammation is suppressed by ICZ treatment

**Figure 4:**
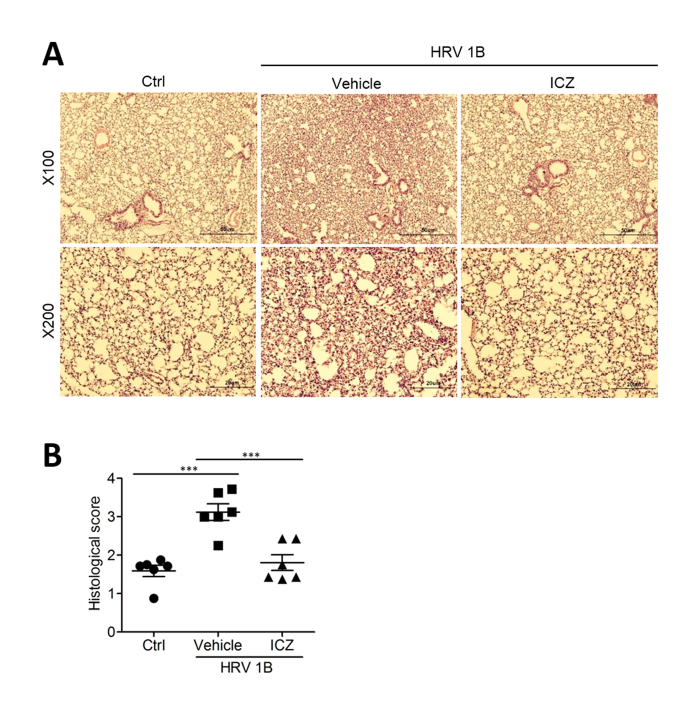
ICZ treatment improves acute lung inflammation caused by HRV1B infection. Lung tissue samples were obtained from HRV1B-infected mice treated with ICZ. (**A**) Lung inflammation was evaluated by hematoxylin and eosin staining. (**B**) Histological scores were assigned according to the presence of edema, hemorrhage, and cell infiltration. Data represent mean ± SEM (n = 6). ^∗∗∗^P < 0.001, Newman-Keuls multiple comparisons test (analysis of variance).

We assessed the histopathological changes in the lungs of mice infected with HRV1B and observed characteristic inflammatory lesions, including necrotizing bronchiolitis and moderate infiltration of immune cells (Fig. 4A). HRV-infected mice treated with ICZ showed mild inflammation with decreased inflammatory cell infiltration, pulmonary edema, and hemorrhage as compared to untreated infected mice (Fig. 4B). These results suggest that the lungs are damaged by acute inflammation following HRV infection, which is mitigated by ICZ treatment.

### Intranasal administration of ICZ protects against HRV infection

**Figure 5:**
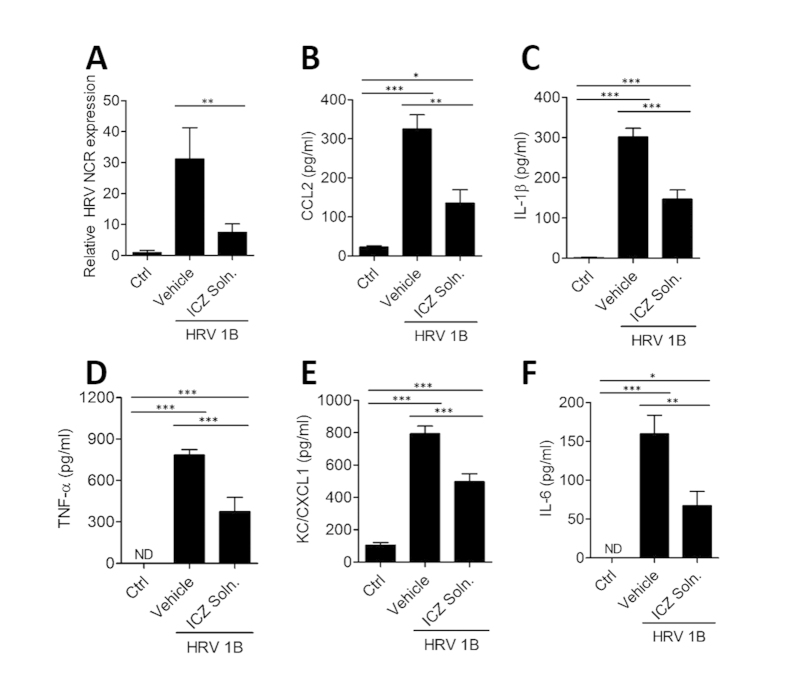
Intranasal administration of ICZ liquid solution prevents HRV infection. Mice were intranasally administered with ICZ liquid solution (ICZ Soln) 1 day before intranasal infection with HRV; 8 h post-infection, lung tissue was homogenized and the supernatant was used for analysis. (**A**) Relative HRV gene expression levels. ^∗∗^P < 0.01 (t test). (**B–F**) Cytokine and chemokine levels. ^∗^P < 0.05, ^∗∗^P < 0.01, ^∗∗∗^P < 0.001, Newman-Keuls multiple comparisons test (analysis of variance).

To determine whether ICZ can act prophylactically against HRV infection, mice were pretreated intranasally with 10 μg ICZ liquid solution 16 h before HRV1B infection. At 8 h post-infection, we confirmed HRV gene expression by real-time PCR and found that HRV1B titer was lower in mice pretreated with ICZ liquid solution as compared to vehicle (Fig. 5A). Moreover, IL-6, CCL2, IL-1β, TNF-α, and CXCL1/KC levels were increased in vehicle-treated infected mice, but were decreased by intranasal pretreatment with ICZ liquid solution (Fig. 5B–F). These results suggest that ICZ has a prophylactic effect against HRV infection.

### Cholesterol dampens the antiviral activity of ICZ against HRV1B

**Figure 6:**
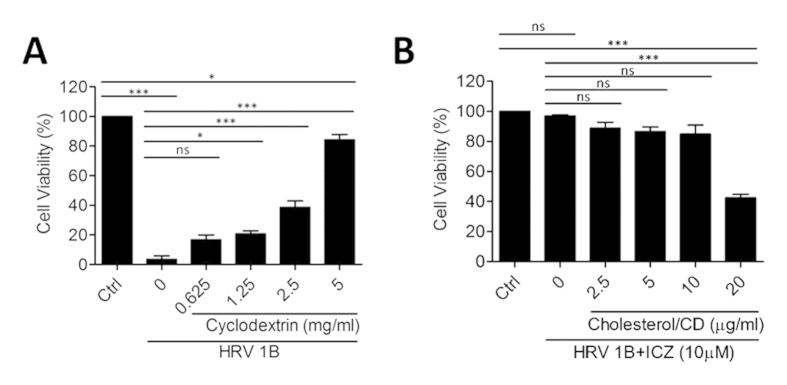
Cholesterol supplementation inhibits the antiviral activity of ICZ. (**A**) HRV1B-infected HeLa cells were treated with CD in the absence of ICZ (**A**). (**B**) Cells were treated with indicated concentrations of cholesterol/CD, followed by 10 μm ICZ. ^∗^P < 0.05, ^∗∗^P < 0.01, ^∗∗∗^P < 0.001, Newman-Keuls multiple comparisons test (analysis of variance).

We speculated that ICZ exerts its antiviral effects by inhibiting cholesterol trafficking, thereby suppressing HRV infection and replication^28^,^29^. To test this hypothesis, we added cholesterol/CD to HeLa cells, which were then infected with HRV1B in the presence or absence of 10 μM ICZ (Fig. 6). Treatment with CD or cholesterol/CD complex with or without ICZ did not affect cell viability in the range of concentrations tested (Fig. S11).

Treatment of cells with 5 mg/ml CD solution prevented HRV infection, possibly by depleting membrane-anchored cholesterol (Fig. 6A). Since cholesterol and CD may have antagonistic roles in the antiviral activity of ICZ, we tested a range of CD concentrations and found that 0.625 mg/ml (0.0625%) CD had no antiviral activity (Fig. 6A) and did not reduce the antiviral activity of ICZ in HRV1B-infected cells (data not shown). Therefore, cholesterol (20 μg/ml) was dissolved in 0.0625% CD in subsequent experiments. At this concentration, cholesterol inhibited the antiviral activity of ICZ and induced cytotoxicity in infected cells (Fig. 6B). We also found that the antiviral activity of ICZ against CVB3 was abrogated in the presence of cholesterol/CD (Fig. S12). These data suggest that the antiviral activity of ICZ is associated with inhibition of cholesterol trafficking to the cell membrane.

### ICZ inhibits cholesterol shuttling between plasma membrane and late endosome/lysosome

To elucidate the mechanism of action of ICZ, we firstly assessed the effect of ICZ on the replication of HRV14 replicon. ICZ effectively inhibited the replication of HRV14 replicon as Rupintrivir, an irreversible inhibitor of the HRV 3C protease^30^, did suggesting that ICZ might not be involved in the inhibition of HRV entry (Fig. S13). Next, we investigated how long the addition of ICZ can be delayed before it loses its antiviral activity in cultured cells using modified time-of-drug addition assay^31^. We found that more than 50% of cells survived when ICZ was added at 0, 1, 2, 4, 6, 8, and 10 h post-infection, but it was more effective when ICZ was treated at the early time points just after HRV infection than 2 h or later (Fig. S14). On the contrary, Rupintrivir protected cells from HRV1B-mediated cytotoxicity even when cells were treated with it 8 h post-infection. Collectively, we found that ICZ is early-stage inhibitor for HRV1B replication, but did not inhibit the entry of HRV1B on target cells.

**Figure 7:**
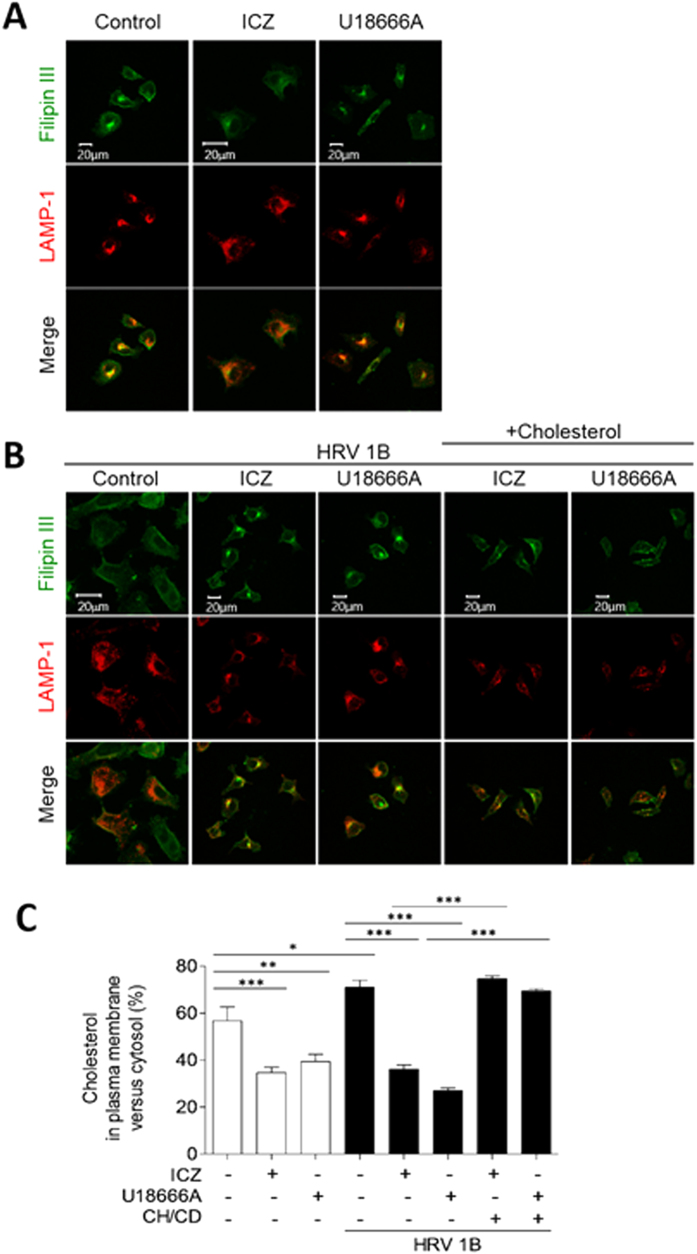
Cholesterol/CD supplementation restores plasma membrane cholesterol levels and suppresses the antiviral activity of ICZ against HRV1B. HRV1B-uninfected (**A**) or -infected (**B**) HeLa cells were treated with vehicle or 10 μm ICZ, and HRV1B-infected cells were supplemented with cholesterol/CD (20 μg/ml cholesterol in 5 mg/ml CD) after ICZ treatment. Cells were fixed and stained for the cholesterol marker filipin III (green) and probed for the endosome marker LAMP-1 (red). Scale bar, 20 μm. (**C**) The percentage of cholesterol in plasma membrane versus cytosol was calculated from (**B**).

To confirm whether ICZ acts as a cholesterol trafficking inhibitor, we stained HeLa cells with filipin III—a histochemical cholesterol marker—and evaluated the expression of the endosome marker lysosome-associated membrane protein (LAMP)-1 (Fig. 7). In uninfected cells, cholesterol was distributed throughout the plasma membrane and in intracellular membranous organelles (Fig. 7A). However, HRV1B infection resulted in the concentration of cholesterol at the plasma membrane, with almost no colocalization with LAMP-1 (Fig. 7B). Treatment of uninfected cells with ICZ or another intracellular cholesterol transport inhibitor (U18666A) restricted cholesterol distribution to some organelles around the nucleus (Fig. 7A), which was also observed in HRV1B-infected cells treated with ICZ (Fig. 7B). We then assessed whether the addition of cholesterol to the culture medium in the form of cholesterol/CD would disrupt the intracellular localization of cholesterol induced by ICZ (Fig. 7B). In presence of excess cholesterol, filipin staining was detected in both cytosolic organelles and the plasma membrane irrespective of HRV1B infection or ICZ treatment. The relative amount of cytosolic cholesterol clustered in intracellular compartments was increased by treatment with ICZ or U18666A (Fig. 7C), while plasma membrane cholesterol content and HRV1B infection rate were reduced. We also found that LAMP-1 colocalized with the cholesterol accumulated around the nucleus after ICZ treatment. These results indicate that ICZ protects host cells by inhibiting cholesterol trafficking to the plasma membrane, which is required for viral replication.

Recent studies have reported that ICZ inhibits mTOR signaling along with endosomal/lysosomal cholesterol trafficking in endothelial cells. We therefore hypothesized that activation of autophagy by ICZ-induced mTOR inhibition may underlie the antiviral activity of ICZ against HRV infection. We treated HRV1B-infected cells with the autophagy pathway inhibitors 3-methyladenine and chloroquine; these failed to inhibit the antiviral activity of ICZ (Fig. S15), suggesting that the mechanism underlying the antiviral activity of ICZ involves inhibition of cholesterol trafficking rather than mTOR inhibition^18^.

## Discussion

HRVs are a major cause of human respiratory tract infection and are specifically responsible for the common cold and exacerbation of asthma^32^. Owing to their heterogeneity and high mutation rates it is difficult to develop vaccines against these viruses, and there are currently no means of preventing or treating HRV infections. However, therapeutic approaches are urgently needed given the high prevalence of infections and recent reports of an association between HRVs and asthma, severe upper respiratory tract infection, and possible complications such as bacterial super-infection.

We identified ICZ as a candidate antiviral agent from a screen of the FDA-approved drug library. ICZ showed antiviral activity not only against HRV but also CVB3 and EV71 in HeLa cells, suggesting that it has broad-spectrum antiviral activity against *Picornaviridae* family viruses. ICZ has been shown to inhibit intracellular cholesterol trafficking and thereby decrease the levels of plasma membrane cholesterol^17^,^18^. Cholesterol-enriched plasma membrane domains have been implicated in HRV infection; indeed, we found that pretreatment of HeLa cells with CD—which depletes membrane cholesterol—inhibited HRV infection. Nystatin and other cholesterol-aggregating reagents reduced echovirus infection in mammalian cells and inhibited the uncoating step of viral particle release by directly regulating alpha2 multivesicular bodies^33^. On the contrary, although inhibition of cholesterol biosynthesis by the treatment of cells with simvastatin, a HMG-CoA reductase inhibitor which can reduce cholesterol and LDL levels, inhibited HRV-mediated chemokine secretion by monocytes and macrophage *in vitro*, the direct antiviral activity of simvastatin against HRV was not shown^28^. However, in our screening results of FDA library to find antiviral drug candidates against HRV, the treatment of cells with several statins including simvastatin and nystatin (10 μM) did not improve the viability of HeLa cells infected with HRV1B (data not shown), suggesting that cholesterol trafficking may be important for HRV replication rather than cell membrane or cytosolic cholesterol content. Thus, cholesterol depletion or restriction of its movement by ICZ may prevent HRV infection; conversely, restoring membrane cholesterol by supplementation suppresses the antiviral effect of ICZ.

A recent study reported that the antiviral activity of ICZ against CVB3 and EV71 is exerted via inhibition of oxysterol-binding protein (OSBP), a master regulator of lipid homeostasis at membrane contact sites between the endoplasmic reticulum and trans-Golgi apparatus^32^ whose exact function is unknown. CVB3 replication takes place in replication organelles in which cholesterol movement is tightly regulated in an OSBP-dependent manner; binding of ICZ to OSBP blocked its cholesterol-shuttling function and consequently inhibited cholesterol trafficking between intracellular membranes, resulting in the accumulation of cholesterol at replication organelles and an enhancement of antiviral activity. Our results are accordance with a recent study^34^; we also extended the investigation of the antiviral activity of ICZ to a murine HRV1B infection model. Our findings indicate that trafficking of endogenous or exogenous cholesterol within cells is critical for HRV replication.

ICZ is an anti-fungal agent that inhibits ergosterol synthesis, which is important for the maintenance of the fungal cell membrane. Although it is considered as having low toxicity, prolonged exposure to high concentrations of ICZ has been associated with serious hepatotoxicity, albeit in rare cases^35^. To reduce the potential risk associated with ICZ administered systemically, we pretreated mice with an ICZ liquid solution via an intranasal route followed by intranasal infection with HRV1B, and found that the ICZ solution effectively prevented HRV infection.

Oral administration of ICZ reduced acute lung inflammation induced by HRV infection by decreasing the levels of pro-inflammatory cytokines and chemokines including IL-6, TNF-α, IL-1β, CXCL1/KC, and CCL2; this was correlated with reduced viral burden in lung tissue by ICZ administration in a mouse model. Importantly, we found that ICZ pretreatment prevented HRV infection in mice. The antiviral effects of ICZ were exerted via inhibition of cholesterol trafficking by ICZ. In conclusion, our findings suggest that ICZ can be used to prevent or treat HRV infection.

## Methods

### Viruses and cell lines

HRV1B, HRV14, and HRV15 were obtained from the American Type Culture Collection (Manassas, VA, USA) and propagated by infection in HeLa cells^36^, which were maintained in minimal essential medium (MEM) supplemented with 10% fetal bovine serum (FBS) and 1% antibiotic-antimycotic solution (Invitrogen Life Technologies, Karlsruhe, Germany). Trypsin-EDTA was purchased from Invitrogen Life Technologies.

### Reagents

The antiviral activity of ICZ against HRVs was detected by *in vitro* screening of the Screen-Well FDA-Approved Drug Library V2 Version 1.0 (BML-2843-0100; Enzo Life Sciences, Lausanne, Switzerland). ICZ and hydroxypropyl-β-cyclodextrin (HP-β-CD) were purchased from Tokyo Chemical Industry Co. (Tokyo, Japan). Cholesterol, Rupintrivir, and Pleconaril was obtained from Sigma-Aldrich (St. Louis, MO, USA). ICZ was dissolved in dimethyl sulfoxide at a concentration of 1 mM and diluted with cell culture medium for *in vitro* experiments. HP-β-CD was dissolved in distilled water at a concentration of 100 mg/ml (10%, w/v). The cholesterol/CD complex solution was prepared by dissolving cholesterol (2.5–20 mg/ml) in methanol by heating and diluting 100 times with HP-β-CD (10%, w/v) solution in distilled water. CD and cholesterol/CD solutions were diluted with cell culture medium to appropriate concentrations for *in vitro* experiments.

### Preparation of ICZ for *in vivo* administration

ICZ was dissolved in phosphate-buffered saline (PBS; pH 7.4) and orally administered to mice at a dose of 20 mg/kg. For intranasal administration, we prepared a liquid solution of ICZ (10 mg/ml in PBS); 10 μl of solution were intranasally administered for a final dose of 1 mg/ml.

### Mice and virus infection

Female BALB/c mice (4 weeks of age) were purchased from SPL Laboratory Animal Company/Koatech Bio (Pyeongtaek, Korea) and were intranasally infected with 1 × 10^8^ TCID_50_/ml HRV1B. Mice were maintained in an experimental facility at the Kangwon National University and all experiments were approved by and performed in accordance with guidelines and regulations of the Institutional Animal Care and Use Committee of Kangwon National University (KW-140811-2).

### Antiviral activity assay

Antiviral activity was assessed by the SRB method according to CPE reduction as previously described^37^. Briefly, 1 day prior to infection, HeLa cells (2 × 10^4^/well) were seeded in a 96-well culture plate (BD Biosciences, San Jose, CA, USA). The next day, the medium was replaced with one containing 30 mM MgCl_2_, 1% FBS, diluted virus suspension containing a 50% cell culture infective dose of virus, and an appropriate concentration of test compound. Cells were incubated at 32 °C in 5% CO_2_ for 2 days until the target CPE was achieved. After incubation in ice-cold 70% acetone for 30 min, cells were stained with 0.4% (w/v) SRB (Sigma-Aldrich) in 1% acetic acid solution. Cell morphology was visualized at 10× and 32× magnifications using an Axiovert 10 microscope (Zeiss, Wetzlar, Germany). Bound SRB was solubilized with 10 mM unbuffered Tris base solution, and the absorbance was read at 562 nm using a VERSAmax microplate reader (Molecular Devices, Palo Alto, CA, USA) with a reference absorbance of 620 nm. The percentage of viable cells was calculated based on measured optical density.

### Cytokine and chemokine assays

Cytokine and chemokine levels were evaluated with ELISA kits for TNF-α, IL-1β, and CCL2 (eBioscience, San Diego, CA, USA) and for CXCL1/KC (R&D Systems, Minneapolis, MN, USA). BALF was obtained from mice as previously described^38^. Lung tissue from mice infected with HRV1B was homogenized, and levels of cytokines and chemokines in the supernatant were evaluated as previously described^39^. The absorbance was read at 450 nm on a Spectra Max 340 instrument (Molecular Devices).

### Real time PCR

Total RNA was isolated using the QIAamp viral RNA Mini kit (Qiagen, Valencia, CA, USA). The reverse transcription reaction consisted of RNase inhibitor, murine Maloney leukemia virus reverse transcriptase with 5 × buffer, oligo(dT) 15 primer, and a dNTP mixture (all from Promega, Madison, WI, USA). Quantitative real-time PCR was carried out using Thunderbird SYBR qPCR Mix (Toyobo, Osaka, Japan) and the CFX96 optics module (Bio-Rad, Hercules, CA, USA) with the following primers: HRV 5′-NCR-up, 5′-TCC TCC GGC CCC TGA ATG-3′ and HRV 5′-NCR-down, 5′-GAA ACA CGG ACA CCC AAA G-3′. To determine and discriminate between the negative-strand and positive-strand HRV1B RNA, AccuPower^®^ Rhinovirus A customized Real Time RT-PCR Kit (Bioneer Inc. Co. Daejeon, Korea) was used.

### Flow cytometry

Cells were collected from BALF and stained with the following antibodies for flow cytometry: fluorescein isothiocyanate-conjugated anti-CD11b, allophycocyanin-conjugated anti-CD11c, phycoerythrin:Cy-7 conjugated anti-Ly 6G, and phycoerythrin-conjugated anti-F4/80 (BD Biosciences). Cells were sorted on a FACSVerse instruments (BD Biosciences) and data were analyzed with BD FACSuite software.

### Histological analysis

Histolopathological changes in lungs of HRV1B-infected mice treated with ICZ were assessed as previously described after modification^40^,^41^. Briefly, the tissue was fixed in 4% formaldehyde (Dana, Seoul, Korea) and dehydrated in a graded series of ethanol, washed in xylene, and embedded in paraffin. Tissue blocks were sectioned at a thickness of 10 μm; sections were stained with hematoxylin and eosin and blindly scored by a pathologist under a light microscope. Pathological change was described by a board certified pathologist, using a system previously described and modified^40^,^42^. The mean score (0–4) was given reflecting the degree of acute lung inflammation after HRV1B infection. Lung sections were evaluated for several indices of inflammation and cellular infiltrates changes in the conducting airway and alveolar. Changes were assessed subjectively and scored blindly on a 0 to 4 scale for severity (absent, minimal, mild, moderate, marked). Features taken into account were the amount of perivascular and peribronchial inflammatory infiltrates, alveolar septal infiltrates, and alveolar luminal inflammatory cell exudates, hemorrhage and edema, and degree of epithelial impairment.

### Cholesterol assay using filipin III

HRV1B-infected or uninfected HeLa cells (2 × 10^3^) were treated with ICZ (10 μM), cholesterol/CD (20 μg/ml), and the cholesterol synthesis inhibitor U18666A (1.25 μM) (included in the cholesterol assay kit from Abcam, Cambridge, UK). Cells were fixed with a cell-based assay fixative solution for 10 min and stained with filipin III for 30–60 min at room temperature according to the manufacturer’s protocol. An antibody against human LAMP-1, a late endosome/lysosome marker, was purchased from Biolegend (San Diego, CA, USA). Alexa Fluor 546-conjugated goat anti-mouse IgG secondary antibody was purchased from Invitrogen Life Technologies. Cells were visualized by confocal microscopy (LSM780 NLO; Carl Zeiss, Jena, Germany).

### Statistical analysis

Differences between groups were assessed with the Student’s t test. Multiple-group comparisons were carried out by one-way analysis of variance followed by the Newman-Keuls test. P values < 0.05 were considered significant at a 95% confidence interval for all analyses.

## Additional Information

**How to cite this article**: Shim, A. *et al*. Therapeutic and prophylactic activity of itraconazole against human rhinovirus infection in a murine model. *Sci. Rep.* **6**, 23110; doi: 10.1038/srep23110 (2016).

## Supplementary Material

Supplementary Information
