# Supplementary material for: Therapeutic and prophylactic activity of itraconazole against human rhinovirus infection in a murine model
Source: Sci Rep. 2016 Mar 15;6:23110. doi: 10.1038/srep23110 (PMC4791555; doi:10.1038/srep23110)
Supplement: Supplementary Information [file srep23110-s1.pdf]

Therapeutic and prophylactic activity of itraconazole against human rhinovirus infection in a murine model

Aeri Shim[1,*], Jae-Hyoung Song[1,*], Bo-Eun Kwon[1], Jeong-Jun Lee[1], Jae-Hee Ahn[1], Yeon-Jeong Kim[2], Ki-Jong Rhee[3], Sun-Young Chang[4], Younggil Cha[1], Yong-Soo Lee[4], Mi-Na Kweon[5], Kwi Sung Park[6], Dong-Eun Kim[7], Sungchan Cho[7], Hyun-Jong Cho[1], & Hyun-Jeong Ko[1]

[1]*College of Pharmacy, Kangwon National University, Chuncheon, South Korea*

[2]*Department of Biomedical Laboratory Science, Yonsei University, Wonju, South Korea*

[3]*College of Pharmacy, Inje University, Gimhae, South Korea*

[4]*College of Pharmacy, Ajou University, Suwon, South Korea*

[5]*University of Ulsan College of Medicine, Asan Medical Center, Seoul, South Korea*

*These authors contributed equally to this work.

***Address for correspondence**

Hyun-Jeong Ko, Ph.D.

Laboratory of Microbiology and Immunology, College of Pharmacy, Kangwon National University, Hyoja-2 Dong, Chuncheon, 200-701, South Korea, Tel: +82-33-250-6923, Fax: +82-33-255-7865, E-mail: hjko@kangwon.ac.kr

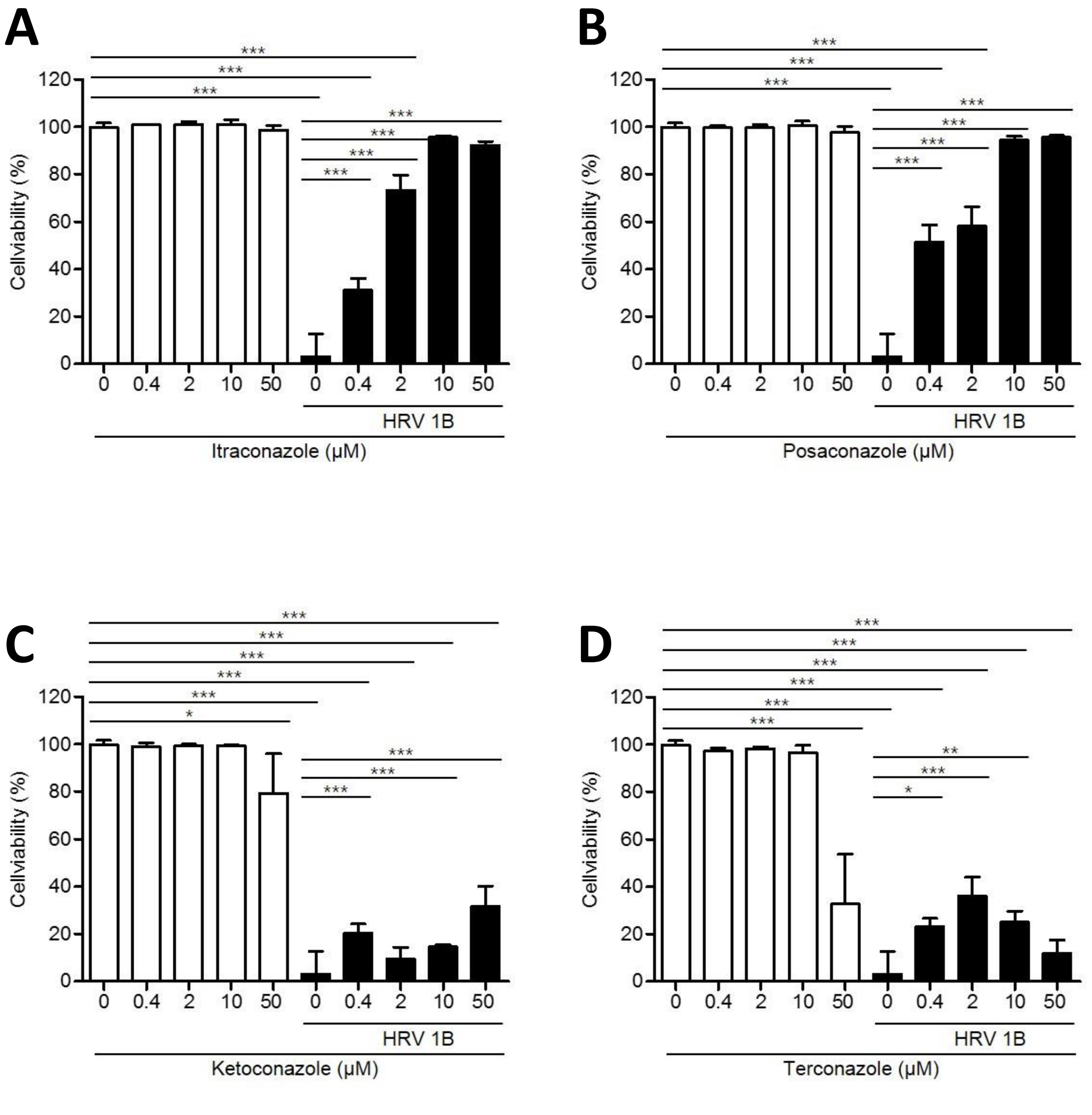

Figure S1. Anti-viral activity of azole family anti-fungal drug against HRV 1B. HeLa cell were treated with (A) ICZ, (B) posaconazole, (C) ketoconazole and (D) terconazole with HRV 1B infection or not. *P<0.05, **P<0.01 and ***P<0.001, Tukey Multiple Comparison Test (ANOVA).

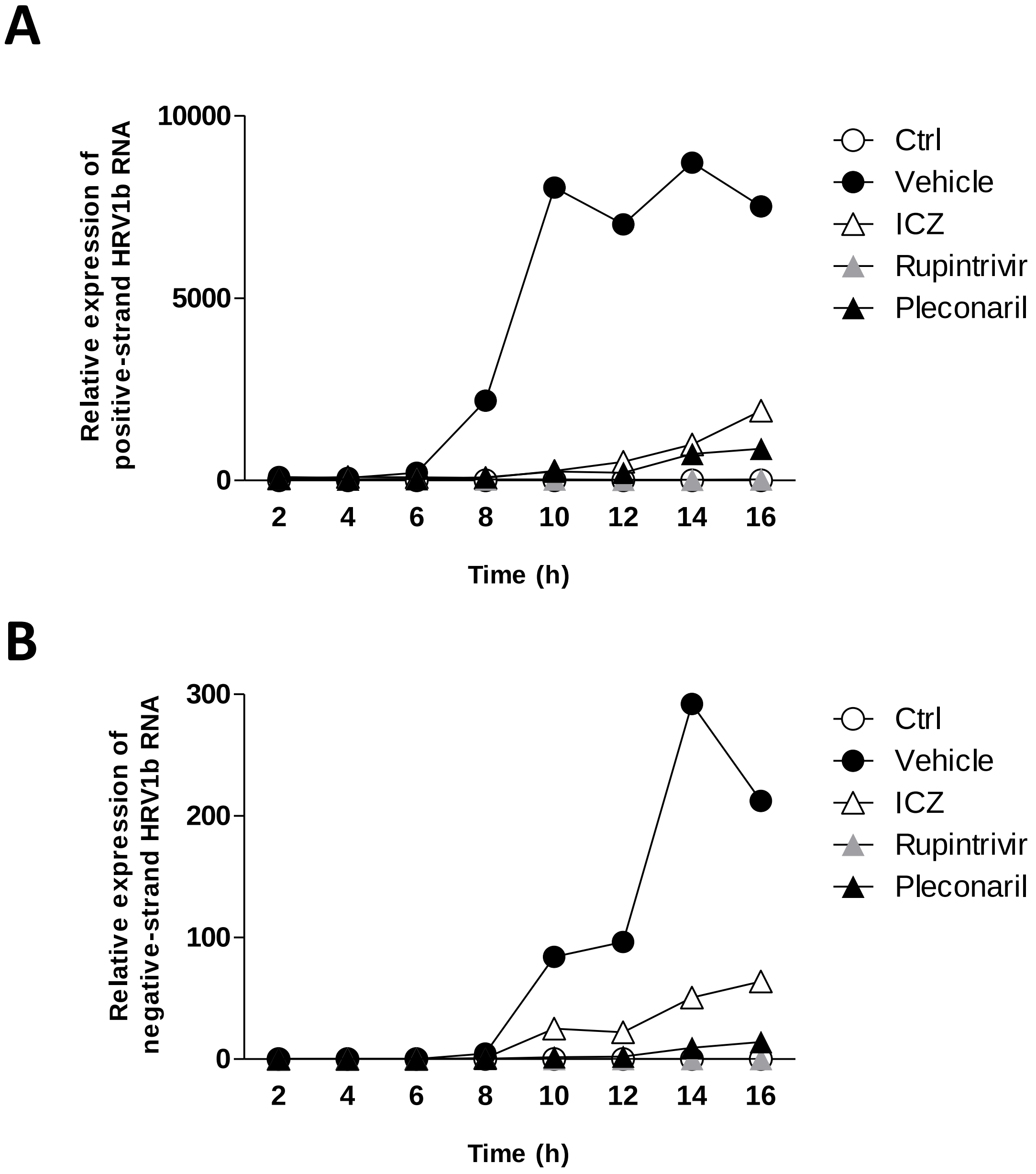

Figure S2. The time course of HRV1B infection experiment.

Hela cells infected with 1 x $CCID_{50}$ HRV1B were harvested at the indicated time points including 2, 4, 6, 8, 10, 12, 14, and 16h post-infection after which 10μM of ICZ, 2μM of Rupintrivir, and 2μM of Pleconaril was added. Total RNA was isolated at the indicated time points post-infection, and the level of positive-strand (A) and negative-strand (B) HRV1B RNA was analyzed by RT-PCR using strand-specific probes (AccuPower® Rhinovirus A customized Real Time RT-PCR Kit, Bioneer Inc. Co.).

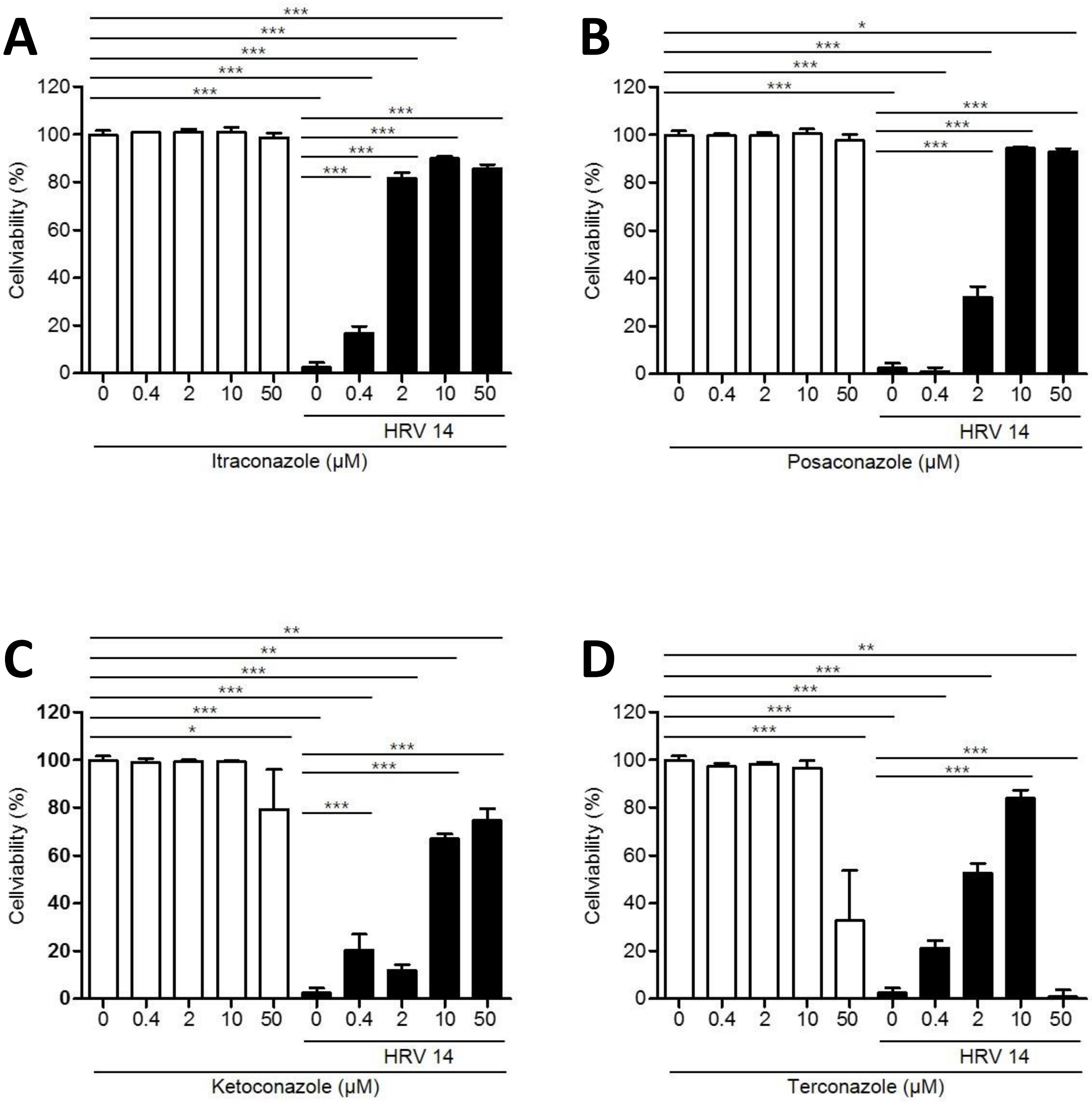

Figure S3. Anti-viral activity of azole family anti-fungal drug against HRV 14. HeLa cell were treated with (A) ICZ, (B) posaconazole, (C) ketoconazole and (D) terconazole, each drug treated in 0.4, 2, 10 and 50 μM with HRV14 infection or not. ICZ has antiviral activity in 2 μM at least concentration to 50 μM. On the other hand, Posaconazole and ketoconazole were shown antiviral activity in 10 μM and 50 μM. Terconazole has antiviral activity in 10 μM only and shown toxicity in 50 μM. *$P<0.05$, **$P<0.01$ and ***$P<0.001$, Tukey Multiple Comparison Test (ANOVA).

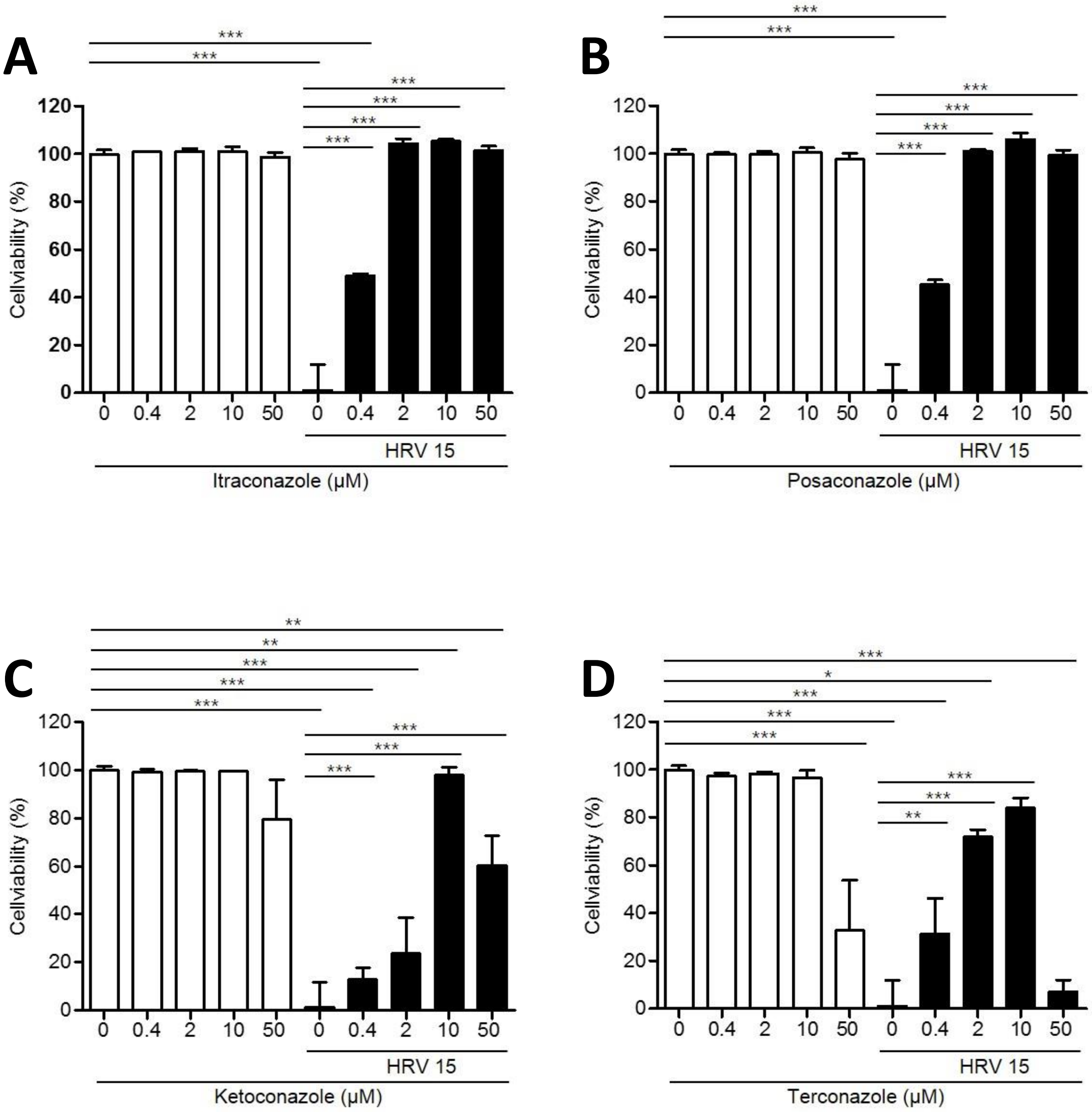

Figure S4. Antiviral effect of anti-fungal drug including ICZ against HRV 15.
HeLa cell were treated with (A) ICZ, (B) posaconazole, (C) ketoconazole and (D) terconazole, each drug treated in 0.4, 2, 10 and 50 μM with HRV15 infection or not. ICZ and posaconazole have antiviral activity in over the 2 μM. Ketoconazole and terconazole were shown only 10 μM concentration and toxicity in 50 μM. *$P<0.05$, **$P<0.01$ and ***$P<0.001$, Tukey Multiple Comparison Test (ANOVA).

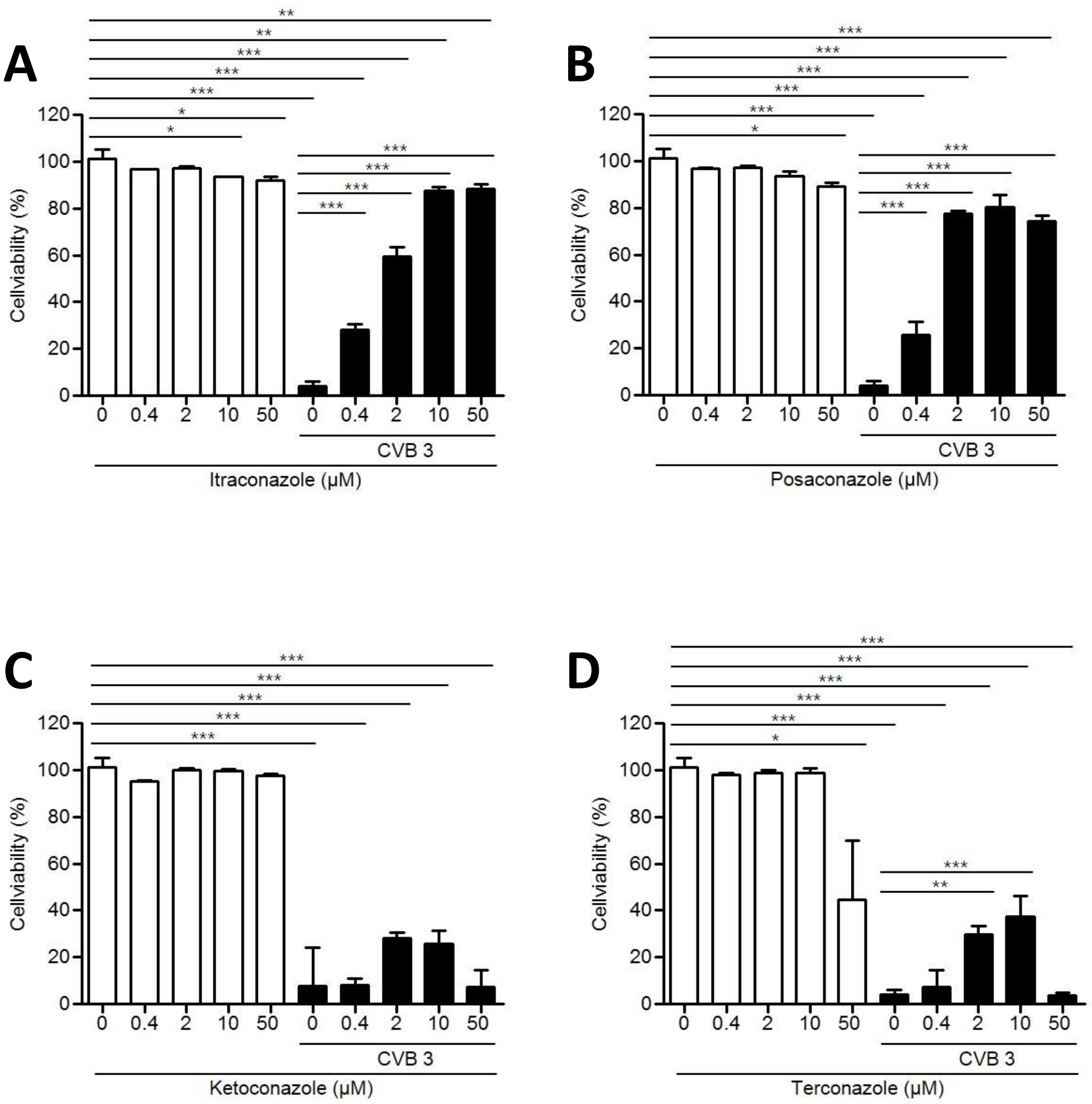

Figure S5. Anti-viral activity of anti-fungal drug against Coxsackie virus B3. Vero cell were treated with (A) ICZ, (B) posaconazole, (C) ketoconazole and (D) terconazole with CB3 infection or not. Posaconazole has over the 80% cell viability at 2μM concentration against CB3. It has antiviral activity better than ICZ. *P<0.05, **P<0.01 and ***P<0.001, Tukey Multiple Comparison Test (ANOVA).

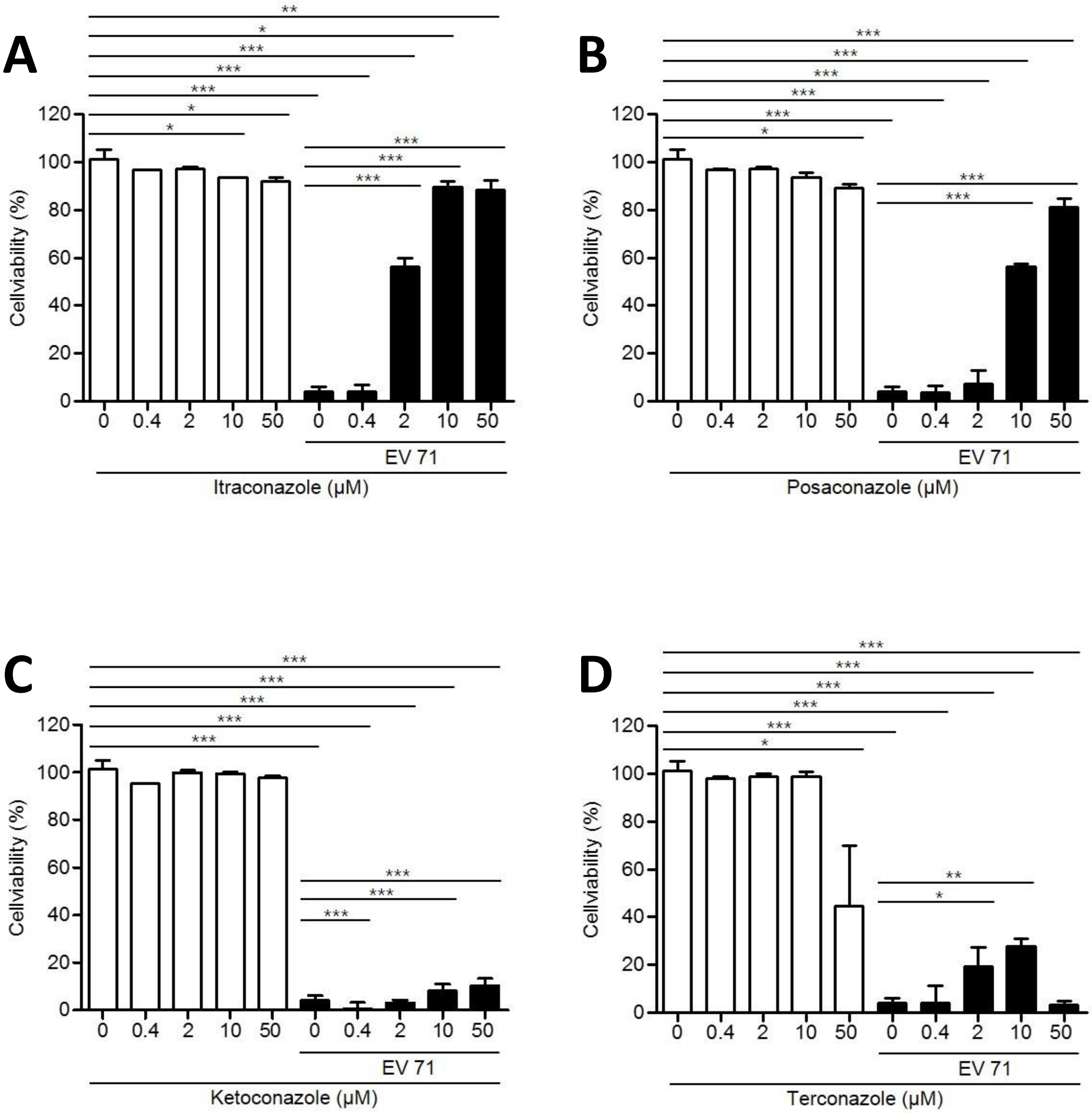

Figure S6. Anti-viral activity of azole family anti-fungal drug against Entero virus 71. Vero cell were treated with (A) ICZ, (B) posaconazole, (C) ketoconazole and (D) terconazole, each drug treated in 0.4, 2, 10 and 50 μM with EV71 infection or not. *P<0.05, **P<0.01 and ***P<0.001, Tukey Multiple Comparison Test (ANOVA).

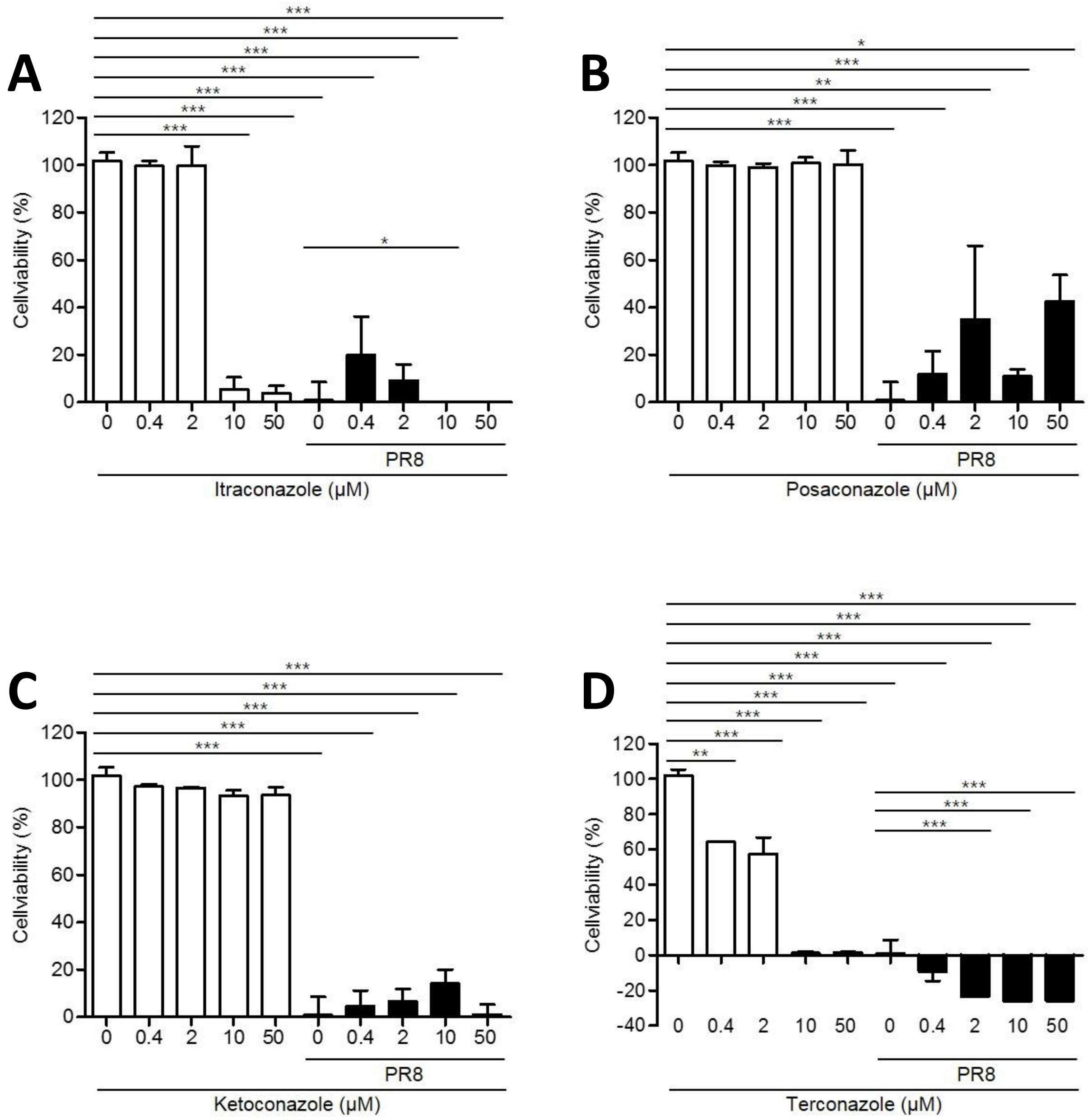

Figure S7. Antiviral effect data of anti-fungal drug including ICZ against PR8. A549 cell were treated with (A) ICZ, (B) posaconazole, (C) ketoconazole and (D) terconazole with PR8 infection or not. Including ICZ, azole type of anti-fungal drug didn't show antiviral activity of influenza A virus. *P<0.05, **P<0.01 and ***P<0.001, Tukey Multiple Comparison Test (ANOVA).

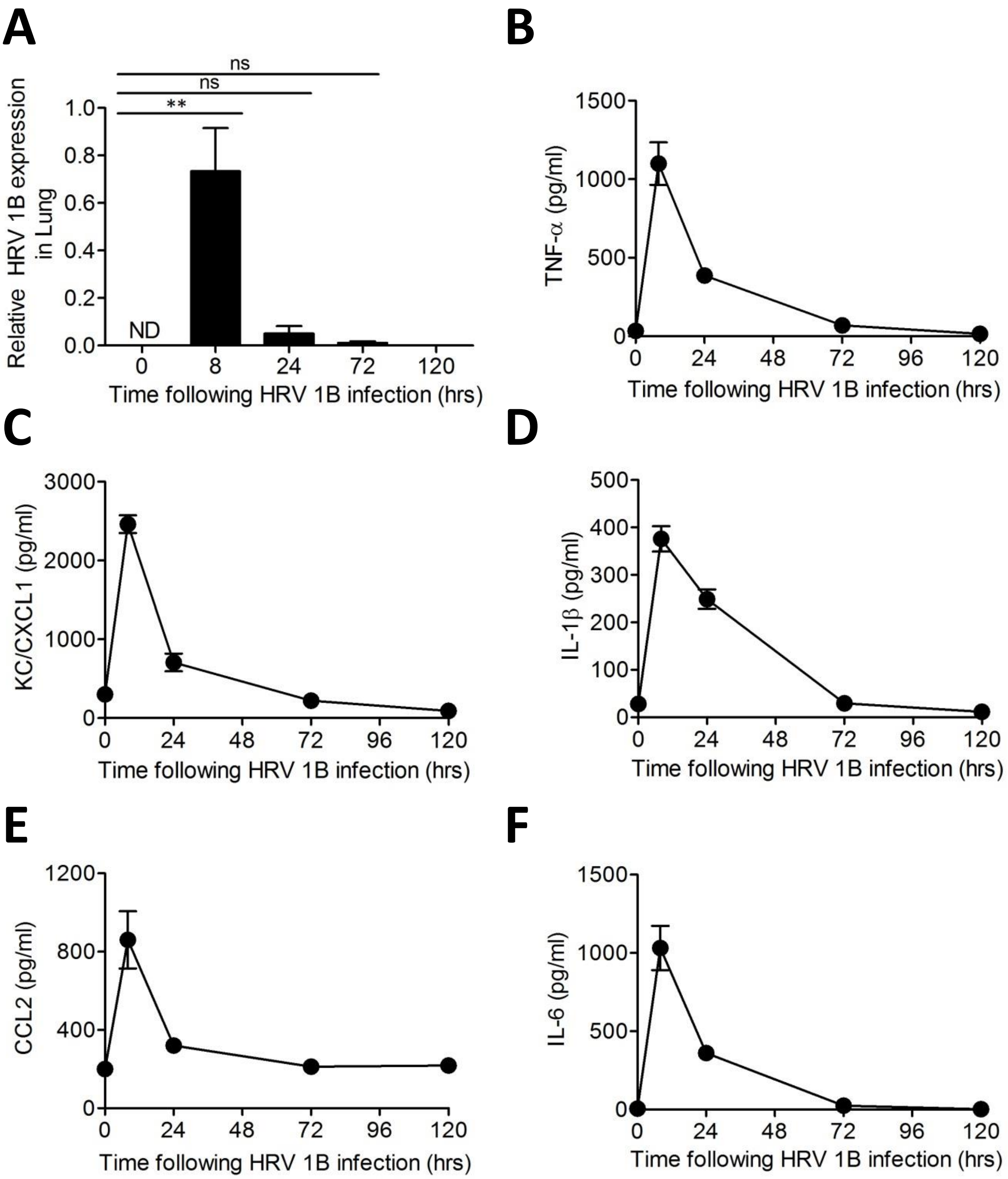

Figure S8. A kinetic analysis of HRV1B infection in mice. HRV1B was intranasally administered into mice, and lung tissues were obtained. Cytokine/chemokine and HRV 1B gene expression was analyzed kinetically at 0, 8, 24, 72 and 120 hrs after infection. The amount of cytokines and chemokines were assessed by ELISA including (A) TNF-α, (B) KC, (C) IL-1β, (D) CCL2 and (E) IL-6. (F) Relative HRV 1B expression was evaluated by real time PCR.

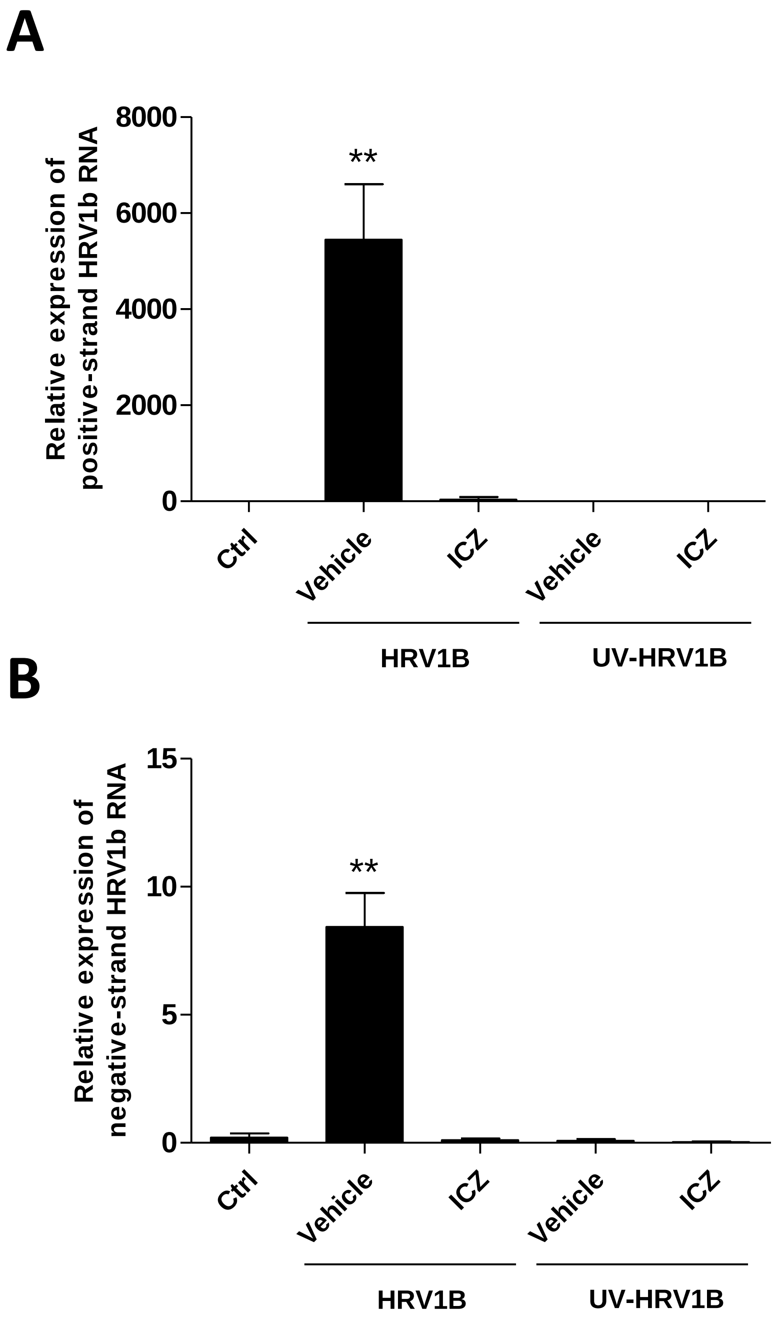

Figure S9. The determination of HRV1B replication *in vivo*.

4 weeks old female BALB/c mice were intranasally infected with 1 × 10^8 $TCID_{50}$/ml HRV1B (three mice per group). Eight hours post-infection, total RNA were isolated from the lung tissues. Mice were infected with either HRV1B or UV-treated HRV1B, and orally administered with 20 mg/Kg ICZ or vehicle 1 h prior to and 4 h after intranasal HRV1B infection. Intracellular viral RNAs including positive-strand (A) and negative-strand (B) HRV1B RNA were detected by RT-PCR using strand-specific probes. **$P<0.01$, Tukey Multiple Comparison Test (ANOVA) as compared with other groups.

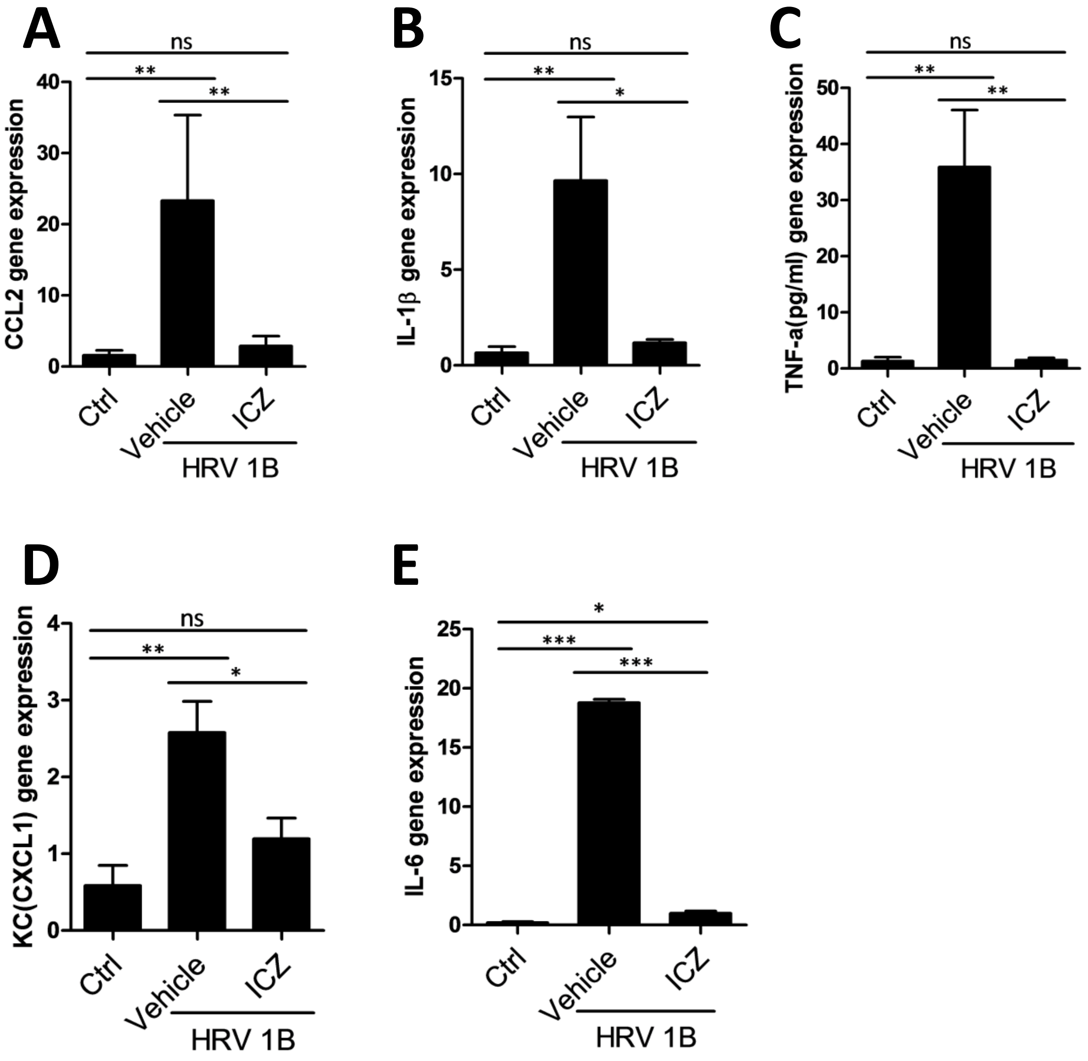

Figure S10. ICZ reduced cytokine/chemokine production induced by HRV1B infection in lung. We assessed the mRNA of cytokines and chemokines in lungs from mice treated with ICZ after HRV 1B infection. We analyzed the levels of (A) CCL2, (B) IL-1β, (C) TNF-α, (D) KC (CXCL1) and (E) IL-6 by real-time PCR. *P<0.05, **P<0.01 and ***P<0.001, Newman-Keuls Multiple Comparison Test (ANOVA).

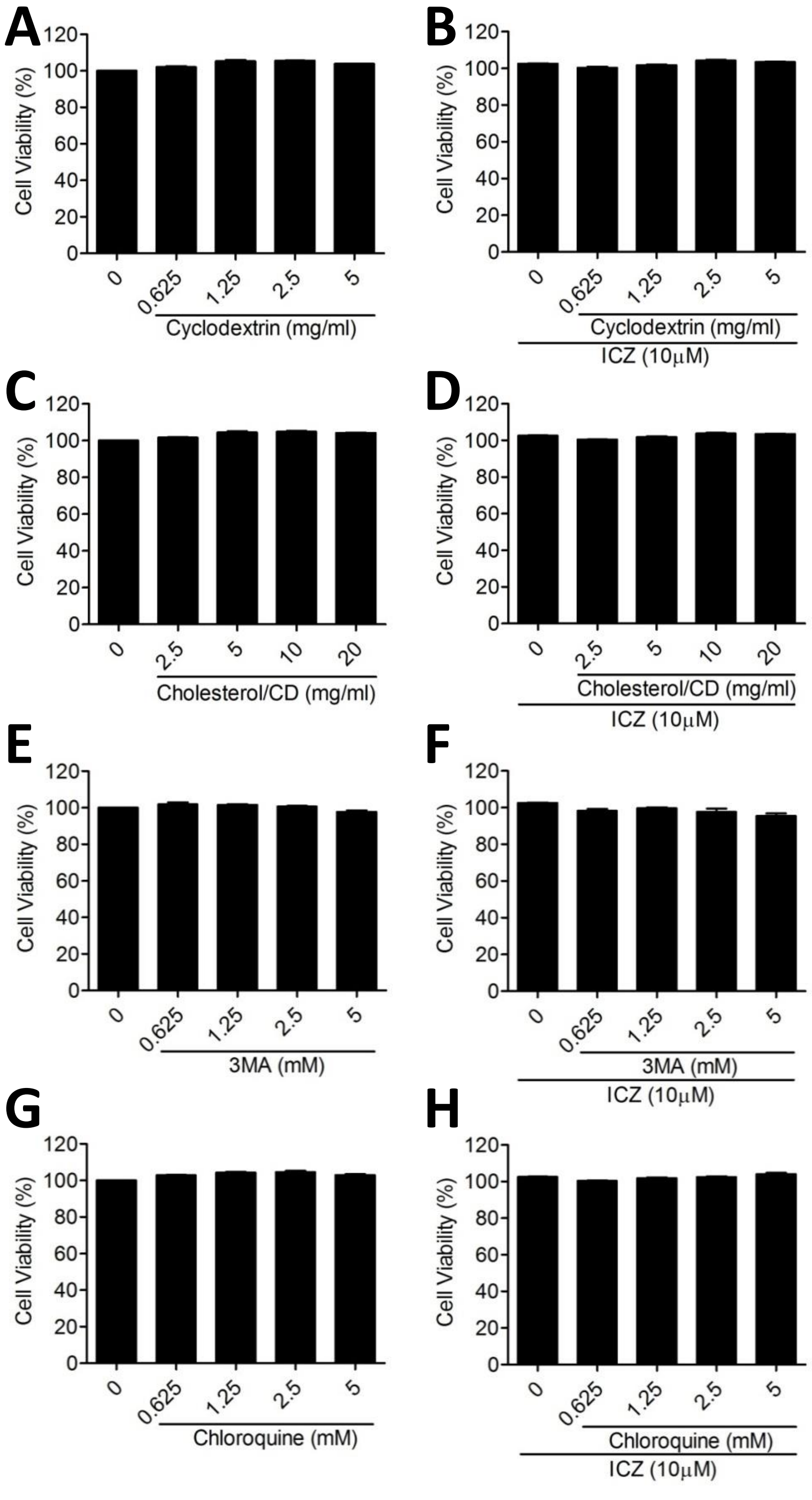

Figure S11. Viability of cells after treatment with cyclodextrin (CD), cholesterol/CD, 3MA, or Chloroquine without HRV1B infection. We confirmed the viability of HeLa cells to check cytotoxicity of cyclodextrin (CD), cholesterol/CD, 3MA, or Chloroquine treatment. Cyclodextrin was treatedinto HeLa cells with vehicle (A) or with ICZ (B). Cholesterol/CD was treated with vehicle (C) or with 10μM ICZ (D). 3MA was treated to HeLa cells with vehicle (E) or with 10μM ICZ (F). Chloroquine was added in HeLa cells culture with vehicle (G) or with 10 μM ICZ (H).

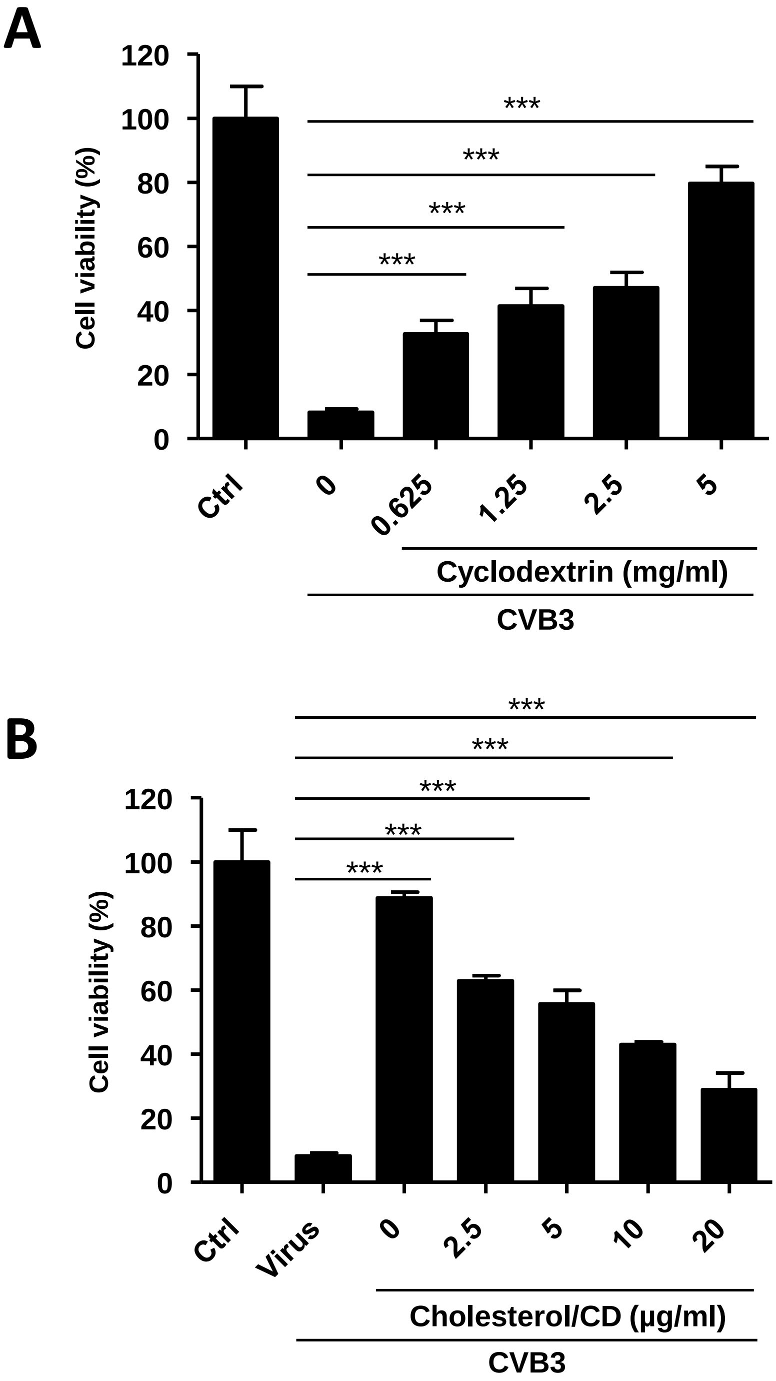

Figure S12. Cholesterol supplement inhibit antiviral activity of ICZ against CVB3 in vitro. To assess the effect of addition of cholesterol/CD or CD would influence on the antiviral activity of ICZ, CVB3-infected Vero cells were treated as indicated. CD was treated in CVB3-infected Vero cells in the absence of 10 μM ICZ (A). (B) Cholesterol/CD was treated at indicated concentrations to HRV1B-infected cells, and 10 μM of ICZ was added. ***$P<0.001$, Newman-Keuls Multiple Comparison Test (ANOVA).

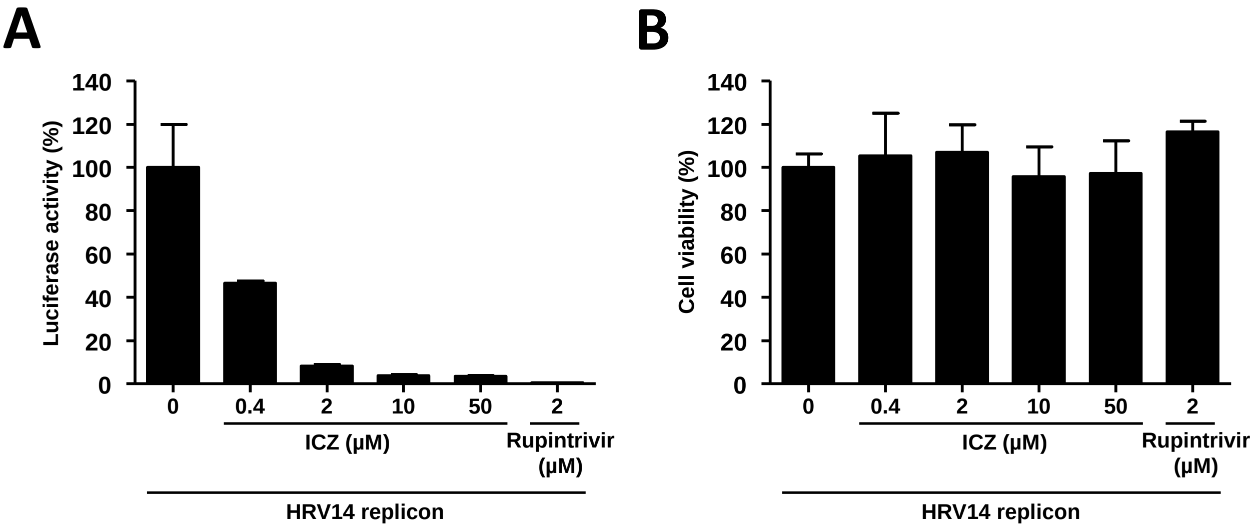

Figure S13. ICZ potently inhibits the replication of the HRV14 replicon.
(A) 293T cells were transfected with in vitro-transcribed HRV14-replicon RNAs, immediately treated with the indicated concentrations of ICZ for 8h, and then assayed for firefly luciferase activity. The luciferase activity of DMSO-treated cells was considered to be 100%. (B) In the same conditions, another set of HRV14 replicon-transfected cells was assayed for cell viability using CellTiter-Glo reagent. The activity of DMSO-treated cells was considered to be 100%.

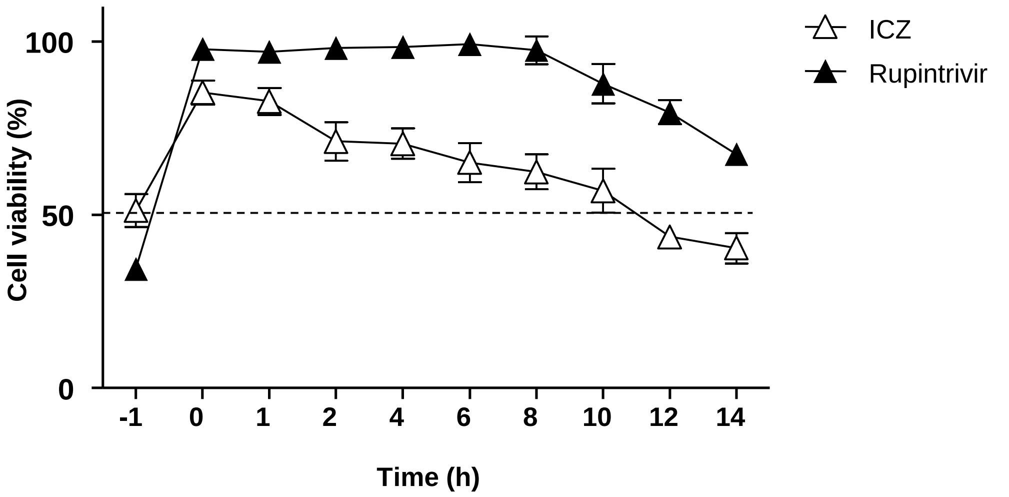

Figure S14. A time-of-addition experiment reveals that ICZ inhibits HRV1B replication at an early stage of infection, but not prior to viral infection.
10μM of ICZ and 2μM of Rupintrivir were added prior to, at the time of, or after viral infection at indicated time points, and percentage of survived cells was analyzed 48h post-infection. HeLa cells treated with drugs prior to viral infection was washed out before infection. Cell viability was evaluated based on results of the SRB assay.

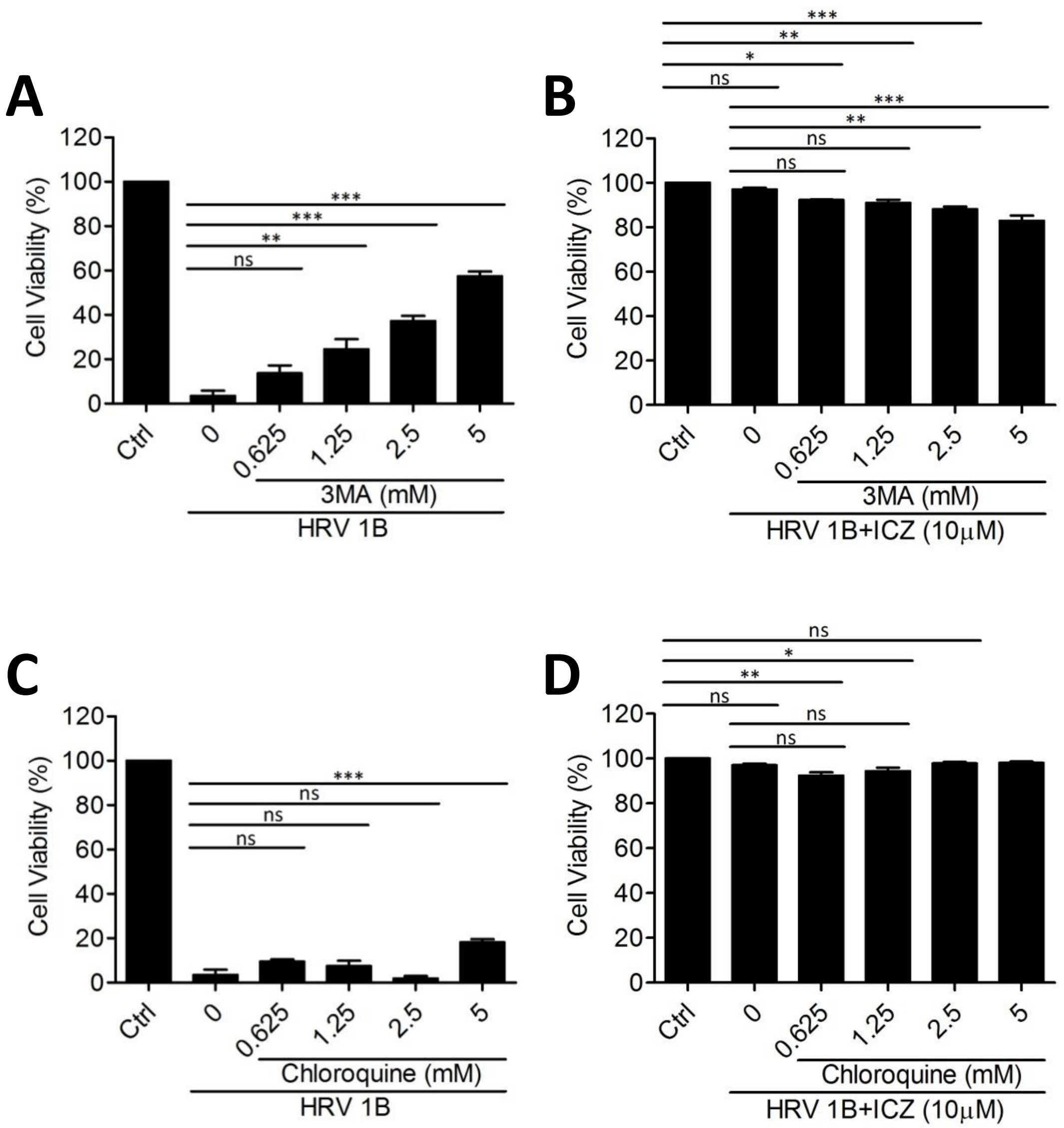

Figure S15. Blockade of autophagy did not attenuate the antiviral activity of ICZ. To assess whether the blockade of autophagy activation may effect on antiviral activity of ICZ, we blocked the autophagy using 3-methyladenine (3MA) and chloroquine. Viability of cells was calculated after treatment of HRV1B-infected HeLa cells with 3MA in the absence (A) or in the presence (B) of 10μM ICZ. HRV1B-infected Cells were treated with chloroquine without (C) or with (D) ICZ treatment.

Supplementary Information for Materials & Methods

Replicon assay

Plasmid pΔP1Luc/VP3 mutant clone, which contain the firefly luciferase gene in place of the P1 capsid-coding region of the HRV14 viral genome, was kindly provided by Stanley M. Lemon (University of North Carolina, USA). HRV14 replicon plasmid linearized by Mlu I was used for *in vitro* RNA transcription with the Ribomax large-scale RNA production system (Promega). The 293T cells (3×10^5 cells/well) in a 6-well plate were transfected with 0.4 mg HRV14 replicon RNAs using Lipofectamine 2000 (Promega), split into 96-well plates (2×10^4 cells/well), and simultaneously treated with various doses of ICZ and Rupintrivir. Twelve hours after treatment, the cells were assayed for firefly luciferase activity using the One-Glo Luciferase Assay System (Promega). Cell toxicity was also measured using CellTiter-Glo Luminescent Cell Viability assays (Promega).
